# *MiR-22*, regulated by MeCP2, suppresses gastric cancer cell proliferation by inducing a deficiency in endogenous S-adenosylmethionine

**DOI:** 10.1038/s41389-020-00281-z

**Published:** 2020-11-10

**Authors:** Dongdong Tong, Jing Zhang, Xiaofei Wang, Qian Li, Liying Liu, Axin Lu, Bo Guo, Juan Yang, Lei Ni, Hao Qin, Lingyu Zhao, Chen Huang

**Affiliations:** 1 Department of Cell Biology and Genetics/Key Laboratory of Environment and Genes Related to Diseases, School of Basic Medical Sciences, Xi’an Jiaotong University Health Science Center, Xi’an, Shaanxi 710061 China; 2 Institute of Genetics and Developmental Biology, Translational Medicine Institute, School of Basic Medical Sciences, Xi’an Jiaotong University Health Science Center, Xi’an, Shaanxi 710061 China; 3 Department of Clinical Medicine, Medical College of Yan’an University, Yan’an, 716000 Shanxi China; 4 Instrument Analysis Center, Xi’an Jiaotong University, 710049 Shaanxi Province, China; 5 Department of peripheral vascular disease, 1st Affiliated Hospital of Xi’an Jiaotong University, 710061 Shaanxi Province, China

**Keywords:** Cancer metabolism, Gastric cancer

## Abstract

This study investigated the effect of methyl-CpG-binding protein 2 (MeCP2) on miRNA transcription. Our results of miRNA chip assay and ChIP-seq showed that *MeCP2* inhibited the expressions of numerous miRNAs by binding to their upstream elements, including not only the promoter but also the distal enhancer. Among the affected miRNAs, *miR-22* was identified to remarkably suppress gastric cancer (GC) cell proliferation, arrest G1–S cell cycle transition, and induce cell apoptosis by targeting *MeCP2*, *MTHFD2*, and *MTHFR*. Understanding GC metabolism characteristics is the key to developing novel therapies that target GC metabolic pathways. Our study revealed that the metabolic profiles in GC tissues were altered. SAM (S-adenosylmethionine), a universal methyl donor for histone and DNA methylation, which is specifically involved in the epigenetic maintenance of cancer cells, was found increased. The production of SAM is promoted by the folate cycle. Knockdown of *MTHFD2* and *MTHFR*, two key enzymes in folate metabolism and methyl donor SAM production, significantly suppressed GC cell proliferation. *MiR-22* overexpression reduced the level of endogenous SAM by suppressing *MTHFD2* and *MTHFR*, inducing *P16*, *PTEN*, and *RASSF1A* hypomethylation. In conclusion, our study suggests that *miR-22* was inhibited by MeCP2, resulting in deficiency of endogenous SAM, and ultimately leading to tumor suppressor dysregulation.

## Introduction

Stomach cancer is the fourth most common malignancy in men and the fifth most common malignancy in women^1^. DNA methylation participates in the regulation of gene expression by recruiting methyl-CpG-binding proteins, such as methylated DNA-binding domain (*MBD*) *1–4* and methyl-CpG-binding protein 2 (*MeCP2*). *MeCP2* amplification and overexpression have been observed in several human cancer types^2–4^. MeCP2 is overexpressed in primary gastric cancer (GC) tissues and is involved in the regulation of GC cell proliferation and apoptosis^3,5^.

Approximately 47% of the investigated human miRNAs have been associated with CpG islands, suggesting that miRNAs are subject to transcriptional regulation by DNA methylation^6^. *MiR-22*, a tumor suppressor gene, is downregulated in a wide variety of tumors, such as colorectal cancer, hepatocellular carcinoma, breast cancer, and lung cancer^7–9^. *MiR-22* is involved in tumorigenesis by targeting *HIF-1α*, *SIRT1*, *CDK6*, *Sp1*, *HDAC4*, *MAX*, *Galectin-9*, *NET1*, *PAPST1*, *ESR1*, *TIAM1*, *cyclin A2*/*CDKN1A*, and *Erbb3*, and its promoter is frequently hypermethylated^10–22^. Topologically associating domains (TADs) facilitate the formation of three-dimensional genomic architecture, providing physical contacts between the genes and the regulatory elements. Chromatin TADs affect the expression of cancer-associated genes by inhibiting or facilitating the interactions between enhancers and promoters^23–26^. We also performed a bioinformatics assay to specifically detect the physical interactions between the enhancer and the promoter of *miR-22* in ESCs and cancer cells.

Folate metabolism, also known as one-carbon metabolism, involves a series of transformations and supports epigenetic maintenance. SAM, a reactive methyl carrier, plays a major role in epigenetics. Methylene tetrahydrofolate reductase (MTHFR) catalyzes the reduction of 5, 10-methyleneTHF (5, 10-mTHF) to 5-methyTHF (5-MTHF) in the cytoplasm. 5-MTHF is the most important naturally occurring form of folate found in organisms. 5-MTHF is converted to tetrahydrofolate (THF) with the transfer of a methyl group to homocysteine to form methionine^27^. Methionine is the substrate for S-adenosyl-methionine synthetase. In addition, in the mitochondria, 5,10-MTHF is regulated by MTHFD2, which generates formic acid through a complicated reaction and is transferred to the cytoplasm for folate metabolism. Folate metabolism is usually altered in cancer^28,29^. Targeted gene analysis indicated that the folate metabolism-related enzymes, MTHFR and MTHFD2, which participate in the formation of the methyl donor SAM, may be novel targets of *miR-22*, suggesting that *miR-22* participates in methyl metabolism. Previous studies have shown that *MTHFR* mutation is closely related to tumor formation^30^, but the expression and molecular mechanism of *MTHFR* in cancer still need to be explored. Silencing the expression of *MTHFD2* inhibited the proliferation of multiple tumors^31–34^ significantly, suggesting that *MTHFD2* is an oncogene. It remains unclear whether *MTHFR* and *MTHFD2* are involved in tumor progression by regulating SAM. SAM is a major sustainer of tumor suppressor genes and histone methylation and has a concentration-dependent effect on the proliferation of colorectal cancer cells^28,29^. A study on network regulation showing that folate metabolism contributes to SAM formation and influences the epigenetics and development of carcinomas is important for developing innovative treatment strategies.

In this study, we aimed to investigate the molecular mechanism by which MeCP2 regulates *miR-22* expression and evaluate the role of *miR-22* in one-carbon metabolism by targeting *MTHFR* and *MTHFD2*.

## Results

### MeCP2 regulates *miR-22* expression by binding to the *miR-22* upstream methylated enhancer

**Fig. 1:**
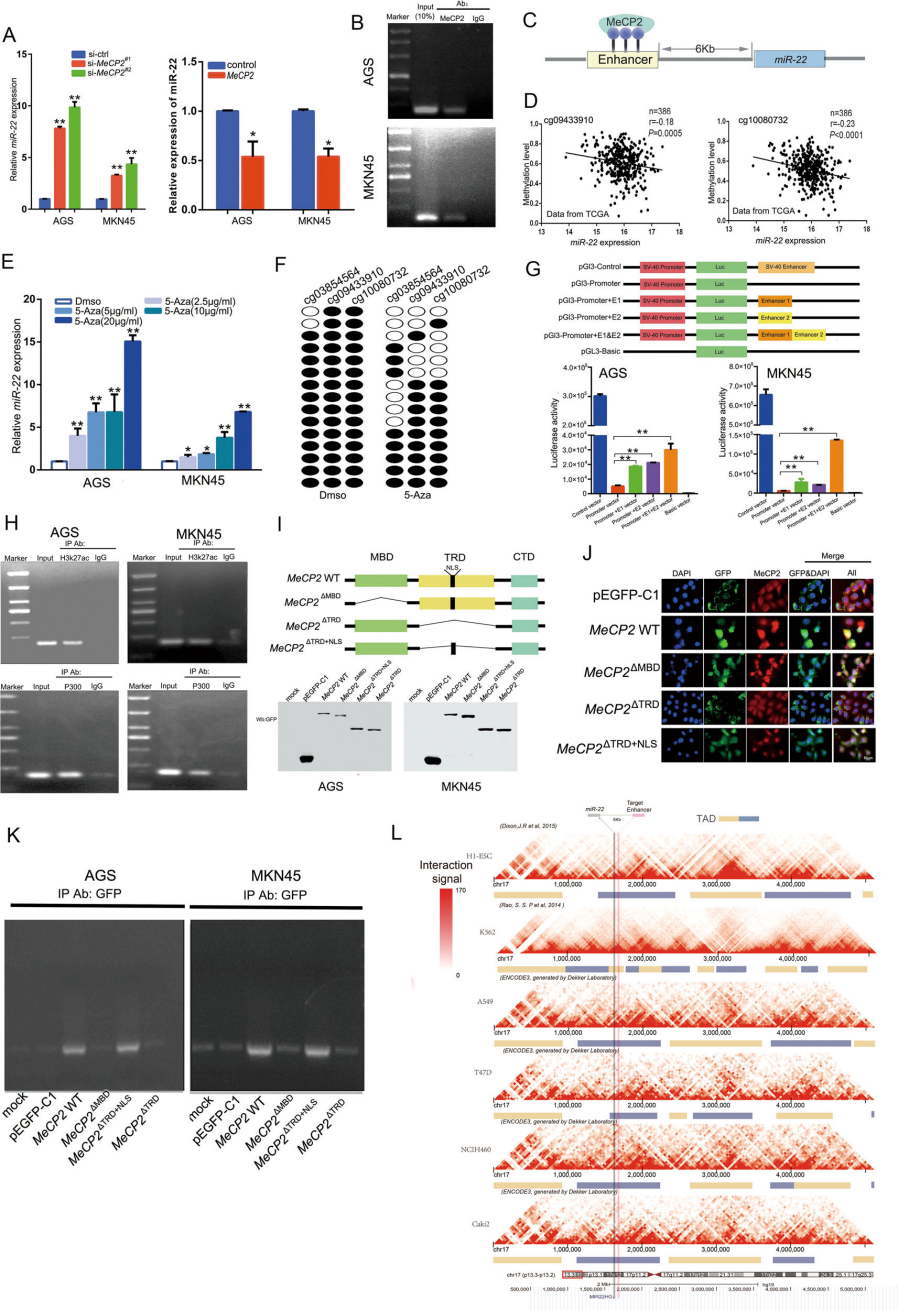
MeCP2 regulates *miR-22* expression via binding to *miR-22* upstream methylated enhancer. **a** *MiR-22* levels in *MeCP2* knockdown or overexpression GC cells. **b** ChIP-PCR assay was used to capture the *miR-22* enhancer region with MeCP2 antibodies in GC cells. **c** Schematic of the relative position of the enhancer and MeCP2-binding site to the *miR-22* location. **d** Correlation between *miR-22* expression and the methylation level of two CpG sites located in the MeCP2 occupied enhancer from the TCGA database (Pearson analysis). **e** *MiR-22* levels in GC cells treated with different concentrations of 5-azacytidine (5-Aza). **f** The methylation levels of CpG sites. Genomic DNA was extracted from DMSO- and 5-Aza-treated AGS cells. *n* = 15, Fisher’s exact test. **g** Schematic of luciferase reporter vectors (top), and luciferase activity of GC cells transfected with the reporter vectors (bottom). **h** ChIP-PCR analysis of the enhancer with anti- H3K27ac, P300, and IgG in AGS and MKN45 cells. **i** Diagram of full-length MeCP2 and different MeCP2 domain deletion vectors (top). After the vectors were transfected into AGS and MKN45 cells, different expression types were detected by western blotting using anti-GFP (bottom). **j** Immunofluorescence of the expression of plasmids MeCP2-WT, MeCP2ΔMBD, MeCP2ΔTRD, and MeCP2ΔTRD+NLS in AGS cells. A GFP antibody was used to label the exogenous proteins, and DAPI was used to label the nuclei. **k** AGS and MKN45 cells were transfected with GFP-tagged MeCP2 full length or domain deletion constructs, and the interaction between MeCP2 and the *miR-22* enhancer was determined by ChIP-PCR. **l** Bioinformatics analysis of Hi-C data. All the results are shown as the mean±SD. *n* = 3, *p < 0.05, **p < 0.01, Student’s *t* test.

To understand the effect of *MeCP2* on the transcription of miRNAs, we performed a microRNA chip assay. The results suggested that many miRNAs, such as *miR-22*, were markedly affected by MeCP2. *MiR-22*, a conserved microRNA, was negatively correlated to *MeCP2* mRNA according to the TCGA data (Supplementary Fig. S1a). To verify whether MeCP2 regulates the expression of *miR-22*, we transfected the *MeCP2* siRNA and the *MeCP2*-overexpressing vector in AGS and MKN45 GC cells. *MeCP2* silencing resulted in *miR-22* upregulation, and the overexpression of *MeCP2* decreased the expression of *miR-22* (Fig. 1a). Next, we performed a chromatin immunoprecipitation (ChIP) sequence assay to uncover the MeCP2-binding sites in the genome, such as the *miR-22* location where MeCP2 bonded an upstream candidate enhancer of *miR-22* (GH17J001721, GeneHancer (GH) Identifier). ChIP-PCR (Fig. 1b) and ChIP-qPCR (Supplementary Fig. S1b) results showed that MeCP2 has binding sites upstream of *miR-22*. In addition, the ChIP-PCR and ChIP-qPCR assays showed low binding of MeCP2 to the enhancer in MeCP2 knockdown GC cells, and high binding in the overexpressed AGS cells (Supplementary Fig. S1c). There are three CpG sites in the enhancer (Fig. 1c), and the TCGA data showed that the methylation levels at two sites (cg09433910 and cg10080732) were negatively correlated with *miR-22* expression (Fig. 1d). We examined ten CpG sites upstream and ten downstream of the MeCP2-binding locations; only the cg09433910 and cg10080732 sites were correlated with *miR-22* (Supplementary Fig. S1d).

To confirm whether DNA methylation is a key modification mechanism in *miR-22* transcription, AGS and MKN45 GC cells were treated with different concentrations of 5-azacytidine (5-Aza). As shown in Fig. 1e, the expression levels of *miR-22* were improved by the methylation inhibitor. Furthermore, bisulfite sequencing showed that the methylation levels of the two CpG sites in the MeCP2-binding location remarkably decreased after treatment with 5-Aza (44.62%) compared with the methylation levels after treatment with DMSO (95.56%) (Fig. 1f). These results suggest that MeCP2-binding site methylation has a dominant role in controlling the transcription of *miR-22*. To verify whether the candidate enhancer serves as a cis-acting regulatory element of the gene activator, we constructed luciferase reporter vectors. In AGS and MKN45 cells, two enhancers were inserted separately into the pGL3.0-promoter vector, and a serial construct showed that these enhancers resulted in an increase in luciferase activity compared to the pGL3.0-promoter, which has no enhancer (Fig. 1g). In addition, we performed a ChIP-PCR assay to verify whether H3K27ac and P300, which have been identified as super-enhancer markers, were binding to this enhancer (Fig. 1h). The ChIP-qPCR results were shown in Supplementary Fig. S1e. To identify the binding domain that accounts for MeCP2 binding to the enhancer, we transfected the expression vectors encoding GFP-tagged *MeCP2* or deletion mutants (*MeCP2*^ΔMBD^, *MeCP2*^ΔTRD^, and *MeCP2*^ΔTRD+NLS^) and control vector to GC cells, and performed immunoprecipitation and immunofluorescence assays with an anti-GFP or anti-MeCP2 antibody to confirm the exogenous gene expression phenotype and subcellular localization (Fig. 1i, j). Subsequently, the results of ChIP with an anti-GFP antibody indicated an MBD, and that the nuclear location of MeCP2 was necessary for the MeCP2 binding of *miR-22* (Fig. 1k, Supplementary Fig. S1f). In order to elucidate the *miR-22* and the enhancer embedded within the same TAD, we analyzed the high-resolution, Hi-C data on ESCs and cancer cells, including K562, A549, T47D, NCIH460, and Caki2 from the GEO public database. The analysis showed that *miR-22* and the enhancer had physical interaction and were positioned within the TAD of 600Kb (Fig. 1l).

### *MiR-22* suppresses proliferation of GC cells by directly targeting *MeCP2*, *MTHFD2*, and *MTHFR* in vitro

**Fig. 2:**
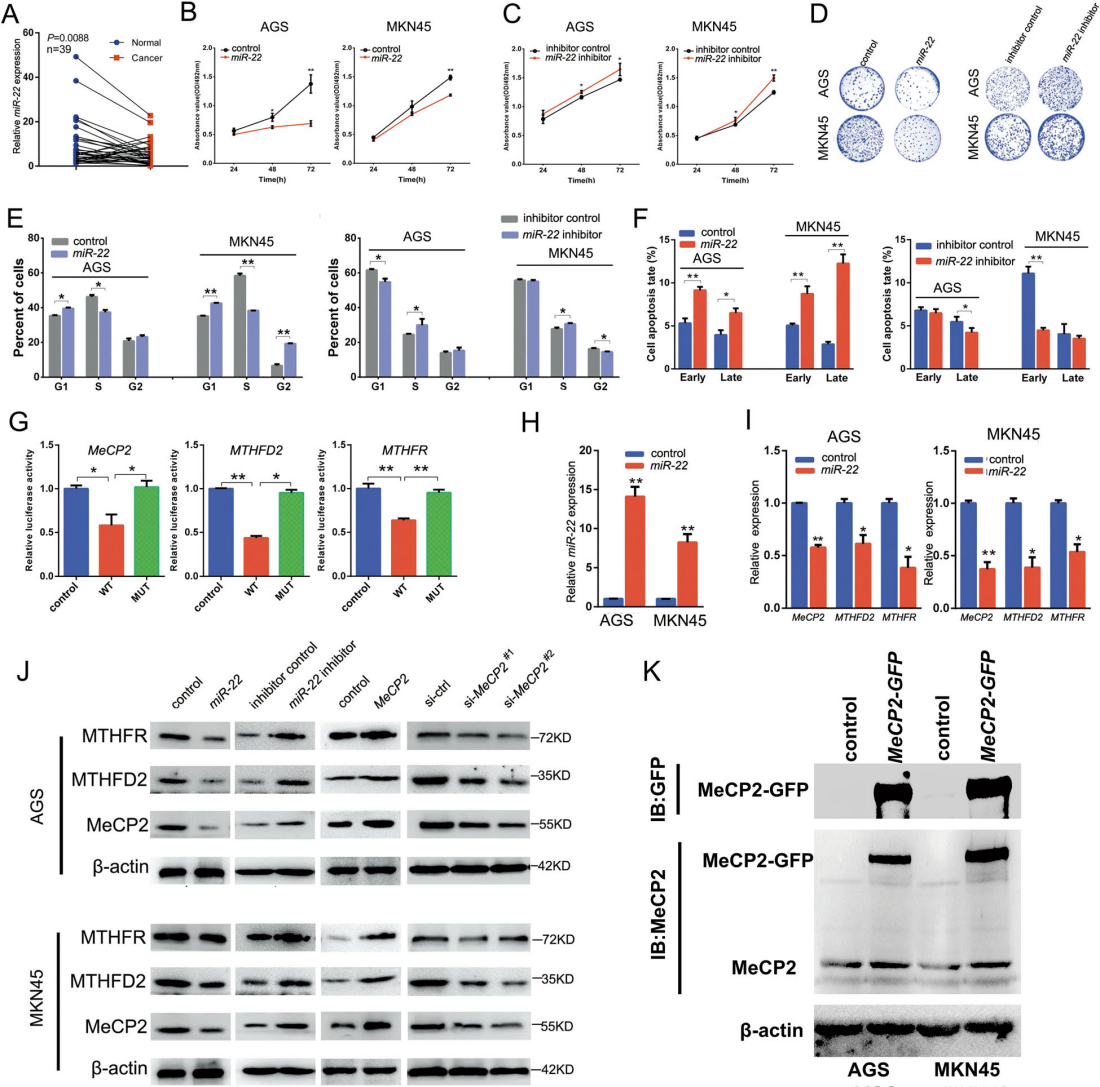
*MiR-22* inhibits AGS and MKN45 cell growth and induced apoptosis by targeting MeCP2, MTHFD2, and MTHFR. **a** Relative expression of *miR-22* in 39 pairs of clinical samples. **b** MTT assay of cell proliferation at 24, 48, and 72 h after transfection with *miR-22* overexpression vector. **c** Cell proliferation was detected after transfection with *miR-22* inhibitor. **d** Colony formation assay 14 days after transfection. **e** Flow cytometry analysis of cell cycle in AGS and MKN45 cells **f** Flow cytometry analysis of cell apoptosis after transfection with *miR-22* overexpression vector or *miR-22* inhibitor. **g** HEK-293T cells were co-transfected with the *miR-22* overexpression vector and indicated reporter vector (control/WT/MUT), and the effects of *miR-22* targeting *MeCP2*, *MTHFD2*, and *MTHFR* were determined by dual fluorescence report assay. **h** Expression of *miR-22* in *miR-22* overexpression vector-transfected AGS and MKN45 cells. **i** Expression of *MeCP2*, *MTHFD2*, and *MTHFR* in AGS and MKN45 cells transfected with *miR-22* overexpression vector. **j** MeCP2, MTHFD2, and MTHFR protein levels were detected after *miR-22* overexpression or inhibition, MeCP2 overexpression or knockdown. **k** Endogenous and exogenous MeCP2 level in GFP-tagged MeCP2 expression vector (MeCP2-GFP) transfected cells. The GFP has a molecular weight of ~27 KD, and the GFP/MeCP2 fusion protein is larger than endogenous MeCP2. All the results are shown as the mean ± SD. *n* = 3, *p < 0.05, **p < 0.01, Student’s *t* test.

We examined *miR-22* expression levels by performing qRT-PCR in 39 matched GC and adjacent tissue samples. Results showed that the expression of *miR-22* appeared downregulated in GC tissues (Fig. 2a). To explore the effect of *miR-22* on GC cell proliferation, we transfected an *miR-22* overexpression vector and miRNA control or *miR-22-3P* inhibitor and control to AGS and MKN45 cells and performed the MTT, clone formation, and flow cytometry assays. Results showed that overexpression of *miR-22* inhibited the proliferation of GC cells (Figs. 2b,d, and Supplementary Fig. S2c) and induced G1/S transition arrest (Fig. 2e) and cell apoptosis (Fig. 2f), whereas inhibition of *miR-22* promoted the proliferation of GC cells (Figs. 2c,d, and Supplementary Fig. S2c) and G1/S transition (Fig. 2e), and suppressed apoptosis (Fig. 2f, Supplementary Fig. S2a and S2b). These results indicated that *miR-22* acts as a tumor suppressor miRNA during GC growth.

Next, we predicted *miR-22* target genes using the TargetScan database. As shown in Supplementary Fig. S2d, *miR-22* could target the 3′-UTR sequence of *MTHFD2* and *MTHFR*. Interestingly, *MeCP2*, which inhibits *miR-22*, was also a candidate target gene of *miR-22*. We constructed wild-type and mutated reporter vectors for three target genes and performed dual-luciferase reporter assays. Results suggest that *miR-22* inhibited *MTHFD2*, *MTHFR*, and *MeCP2* by binding to the 3′-UTRs (Fig. 2g). Further analysis revealed that *miR-22* overexpression suppressed the three target genes (Fig. 2h, i). A western blot analysis was performed to verify whether *miR-22* inhibited the protein expression of the three target genes. As MeCP2 regulated *miR-22* expression at the transcriptional level, MeCP2 silencing decreased MTHFD2 and MTHFR expression, and the overexpression of MeCP2 promoted the expression of the two target genes (Fig. 2j). Based on the above evidence, we concluded that there is a feedback loop between *miR-22* and *MeCP2*. To verify this mechanism, the GC cells were transfected with a *GFP*-tagged *MeCP2* overexpression vector. Western blotting showed that endogenous MeCP2 was increased in *MeCP2-GFP* vector-transfected cells (Fig. 2k).

### *MTHFD2* knockdown suppresses GC cell proliferation

**Fig. 3:**
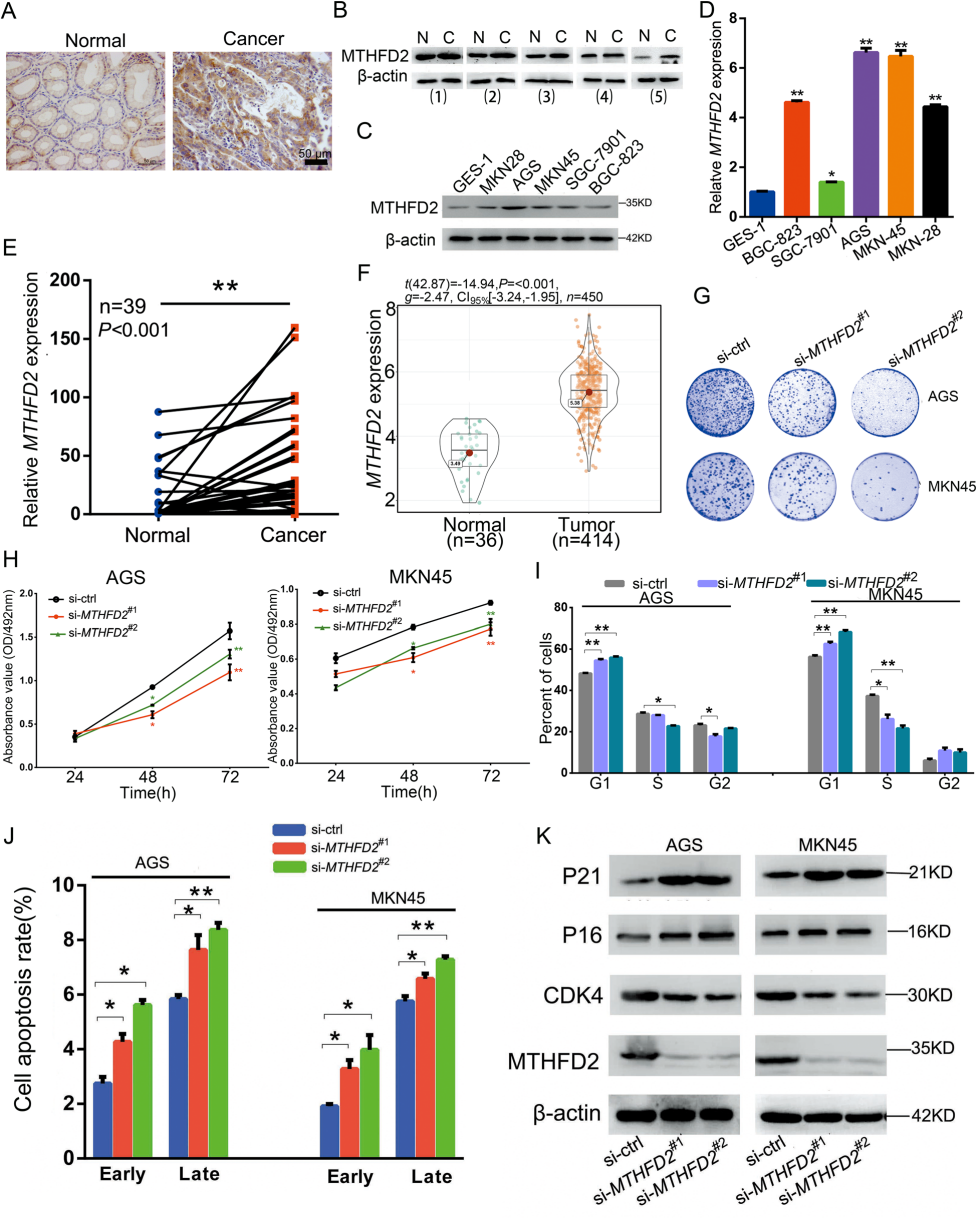
MTHFD2 facilitates GC cell proliferation. **a** IHC staining of MTHFD2 in clinical samples. **b** MTHFD2 protein levels in clinical samples. **c** MTHFD2 protein levels in GC cell lines and normal (GES-1). **d** *MTHFD2* mRNA levels in GC cell lines and normal (GES-1). **e** *MTHFD2* mRNA levels were detected by qRT-PCR in GC tissues and para-carcinoma tissues. **f** Bioinformatics analysis of *MTHFD2* expressions in GC and para-carcinoma tissues based on TCGA data. **g** Colony formation assay 14 days after transfection with *MTHFD2* siRNAs. **h** Cell proliferation was examined by MTT assay at 24, 48, and 72 h after transfection with MTHFD2 siRNAs. **i** Effect of silencing *MTHFD2* on the GC cells cycle. **j** Effect of silencing *MTHFD2* on GC cells apoptosis. **k** Effect of silencing *MTHFD2* on cell cycle-related proteins. All the results are shown as the mean ± SD. *n* = 3, *p < 0.05, **p < 0.01, Student’s *t* test.

To verify the tumor-promoting effect of MTHFD2 on GC, we first examined the expression of MTHFD2 in GC tissues and cell lines. The results showed that MTHFD2 protein and mRNA levels were increased in tumors and cancer cell lines (Fig. 3a–f). Because MTHFD2 was upregulated in GC cell lines, we knocked down *MTHFD2* in the two lines with the highest expression. Results revealed that the silencing of *MTHFD2* decreased the proliferation activity (Fig. 3g,h), blocked the cell cycle G1/S transition (Fig. 3i), and induced apoptosis in GC cells (Fig. 3j and Supplementary Fig. S3). In addition, western blot analysis verified the effect of silencing *MTHFD2* on cell cycle-related proteins. Results showed that silencing of MTHFD2 increased P21 and P16 and decreased CDK4 (Fig. 3k). These findings indicated that MTHFD2 promoted cyclin-related protein expression, induced cell cycle transformation, and facilitated GC cell proliferation.

### *MTHFR* knockdown suppresses GC cell growth

**Fig. 4:**
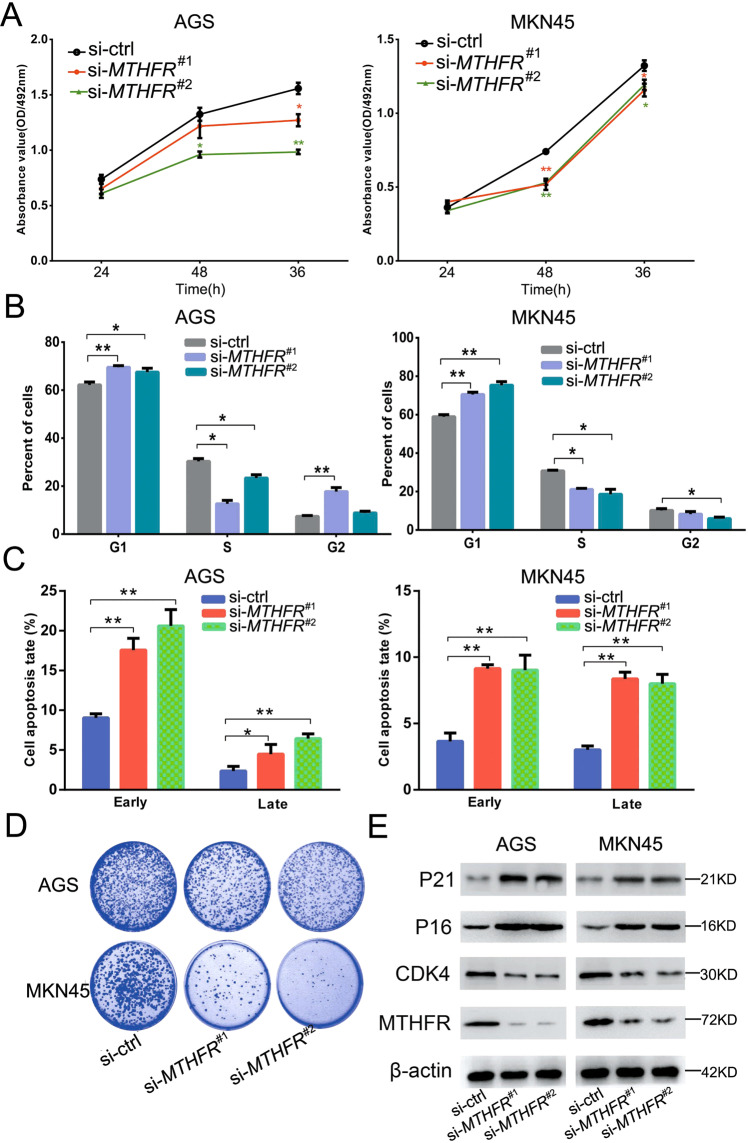
MTHFR promotes GC cell proliferation. **a** MTT assay of cell proliferation at 24, 48, and 72 h after transfection with *MTHFR* siRNAs. **b** Flow cytometry of cell cycle, visualized by PI staining. **c** Cell apoptosis by flow cytometry, visualized using Annexin-V/PI staining. **d** Colony formation assay 14 days after transfection. **e** Effect of *MTHFR* knockdown on cell cycle-related proteins. All the results are shown as mean±SD. *n* = 3, *p < 0.05, **p < 0.01, Student’s *t* test.

To explore whether *MTHFR* dysregulation affects GC cell growth, we transfected the *MTHFR* siRNA to AGS and MKN45 cells. Through cell viability, flow cytometry, and clone formation assays, we found that *MTHFR* silencing could suppress GC cell proliferation and cell cycle G1/S phase transformation and induce cell apoptosis (Fig. 4a–d and Supplementary Fig. S4). To analyze the molecular mechanism of *MTHFR*, western blot analysis was used to detect the effect of *MTHFR* silencing on downstream proteins. The results showed that MTHFR affected GC cell growth by regulating the cell cycle-related proteins, such as P21, P16, and CDK4 (Fig. 4e).

### Overexpression of *MeCP2*, *MTHFD2*, and *MTHFR* eliminates the effects of *miR-22* on GC cells

**Fig. 5:**
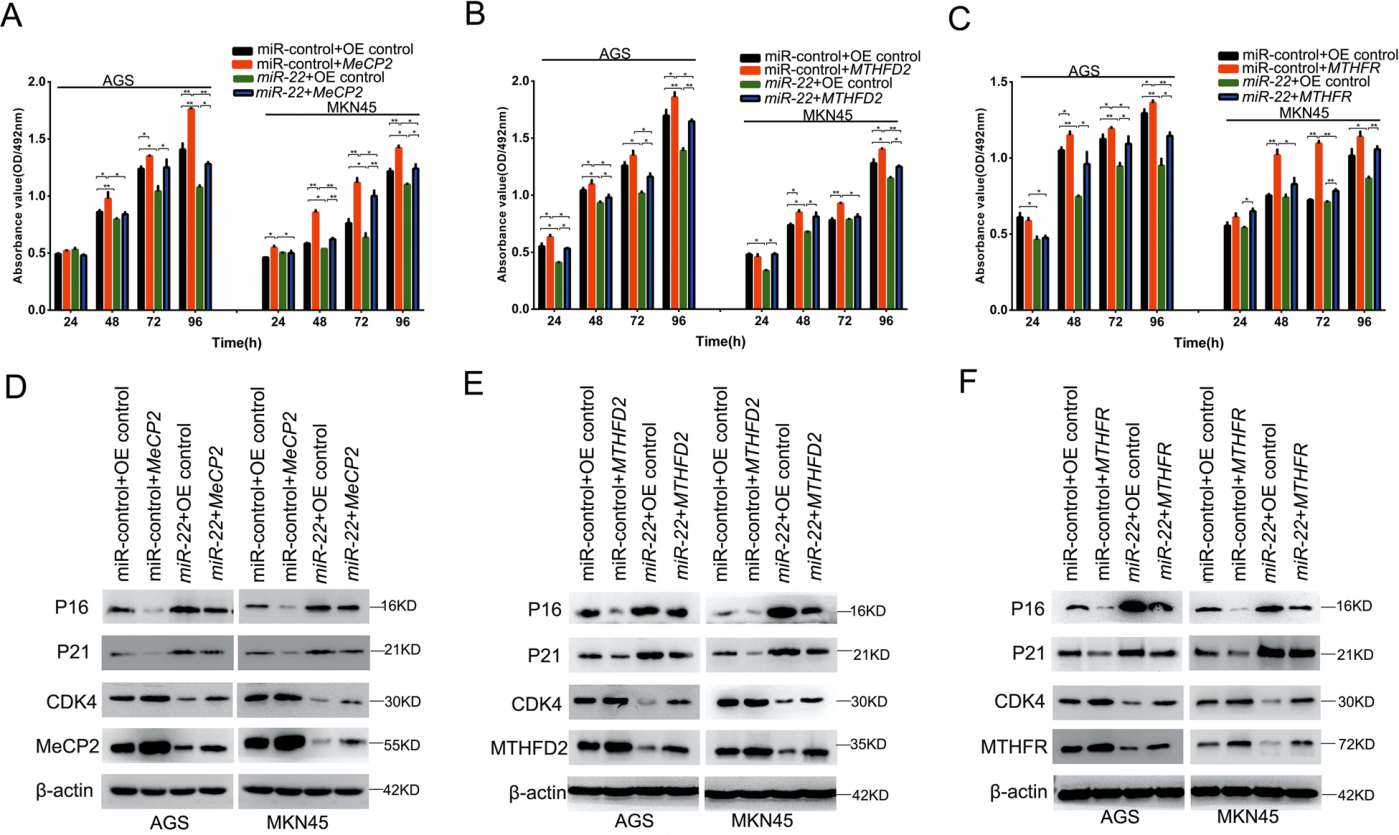
Overexpression of MeCP2, MTHFD2, and MTHFR rescues *miR-22*-induced cellular phenotypes in GC cells. **a** MTT assay of cell proliferation in AGS and MKN45 cells co-transfected with *MeCP2* and *miR-22*. **b** Cell proliferation in AGS and MKN45 cells co-transfected with *MTHFD2* and *miR-22*. **c** Cell proliferation in AGS and MKN45 cells co-transfected with *MTHFR* and *miR-22*. All the results are shown as the mean±SD. *n* = 3, *p < 0.05, **p < 0.01, Student’s *t* test. **d** Expression CDK4, P21, P16, and MeCP2 in cells co-transfected with *MeCP2* and *miR-22*. **e** Expression CDK4, P21, P16, and MeCP2 in cells co-transfected with *MTHFD2* and *miR-22*. **f** Expression CDK4, P21, P16, and MeCP2 in cells co-transfected with *MTHFR* and *miR-22*.

As described above, *miR-22* directly suppressed the expression of *MeCP2*, *MTHFD2*, and *MTHFR*. To clarify their regulatory relationships, rescue experiments were performed, where the three target genes were overexpressed to reverse the effects of *miR-22* on cell proliferation and CDK4, PTEN, P21, and P16 expression (Fig. 5a–f). Our findings indicated that *miR-22* inhibits GC cell proliferation by directly targeting *MeCP2*, *MTHFD2*, and *MTHFR*. As MeCP2 is a *miR-22* epigenetic suppressor, the overexpression of MeCP2 could partly rescue the *miR-22* overexpression effect on MTHDF2 and MTHFR (Supplementary Fig. S5a). Moreover, TCGA data analysis showed that there was a significant correlation between the three genes in GC tissues (Supplementary Fig. S5b).

### *MiR-22* causes SAM defects and promotes the expression of tumor suppressor genes by inhibiting methylation

**Fig. 6:**
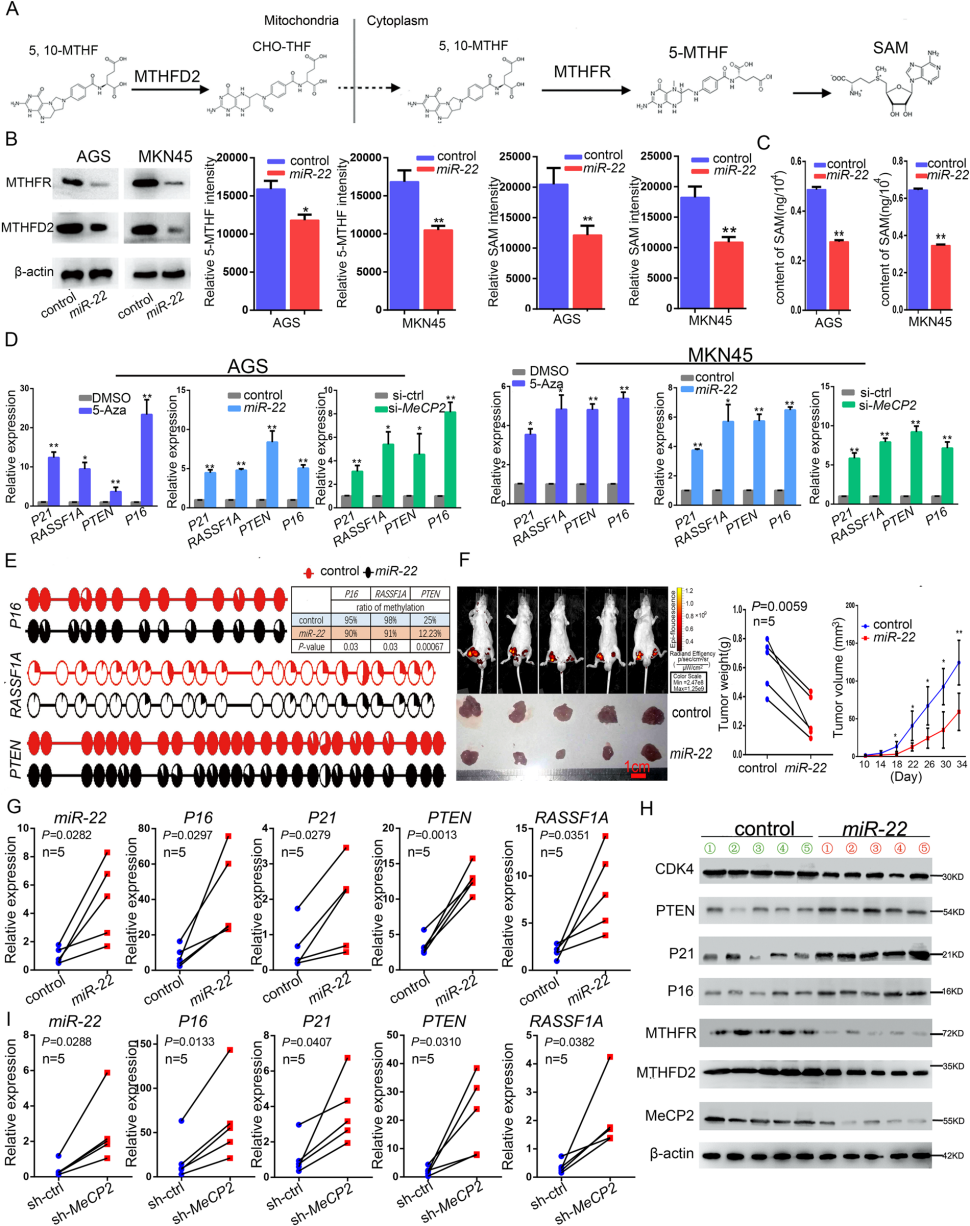
*MiR-22* promotes the expression of tumor suppressor genes by inhibiting SAM synthesis and methylation. **a** The metabolic flux of SAM. **b** 5-MTHF and SAM levels were measured by LC-MS after MKN45 cells transfection with *LV-miR-22*. **c** SAM levels were examined by ELISA after MKN45 cells transfection with *LV-miR-22*. **d** Relative expression of *P16*, *PTEN*, *P21*, and *RASSF1A* in Aza-treated, *miR-22* overexpression and *MeCP2* knockdown GC cells compared with that in the respective controls. **e** The methylation levels of *P16*, *RASSF1A*, and *PTEN* promoter regions were analyzed by BSP in MKN45 cells. The results are shown from the Fisher’s exact test, *n* = 10. **f** Small animal imaging of tumor-bearing mice and gross morphology of xenograft 34 days after injection (left); tumors weight at 34 day after initial injection (middle); growth curve of tumors (right). **g** qRT-PCR of *MiR-22*, *P16*, *PTEN*, *P21*, and *RASSF1A* expressions in *miR-22* overexpression tumor xenografts. **h** The protein levels of CDK4, PTEN, P21, P16, MTHFR, MTHFD2, and MeCP2 in vivo. **i** qRT-PCR of *MiR-22*, *P16*, *PTEN*, P21, and RASSF1A expressions in *MeCP2* knockdown tumor xenografts. The results are shown as the mean±SD. *n* = 3, *p < 0.05, **p < 0.01, Student’s *t* test.

In the mitochondria, 5,10-mTHF is regulated by MTHFD2. 5,10-mTHF is converted to formic acid through complicated reactions, and formic acid is transferred to the cytoplasm for folate metabolism. In the cytoplasm, 5, 10-mTHF is catalyzed by MTHFR to 5-MTHF. 5-MTHF participates in the methionine cycle and forms SAM after a few reaction steps (Fig. 6a). To examine whether *miR-22* can cause the absence of SAM; whether tumor suppressor gene methylation decreases; and whether cell growth is inhibited, the levels of 5-MTHF and SAM were measured using LC-MS and ELISA methods. The results show that 5-MTHF and SAM decreased owing to the overexpression of *miR-22* (Fig. 6b, c). The effect of methylation inhibitor, *miR-22* overexpression, and *MeCP2* knockdown on classic tumor suppressor genes was examined by performing qRT-PCR. *P16*, *PTEN*, and *RASSF1A* are regulated by DNA methylation, whereas *P21* is mainly suppressed by histone hypermethylation. The results showed that the expression levels of *P16*, *PTEN*, *RASSF1A*, and *P21* increased (Fig. 6d). Next, the results of bisulfite sequencing PCR (BSP) directly demonstrated that *miR-22* overexpression decreased the methylation levels of the *P16*, *RASSF1A*, and *PTEN* promoter regions (Fig. 6e). These results suggest that *miR-22* could inhibit endo-SAM, reduce tumor suppressor gene methylation, and lead to expression activation. In vivo, we tested the effect of *miR-22* on MKN45 cell growth. Results showed that overexpression of *miR-22* suppressed the growth of GC (Fig. 6f). Next, we determined the expression levels of *miR-22*, *P16*, *PTEN*, *P21*, and *RASSF1A* in the xenografts, and the results showed an increasing trend consistent with the in vitro results (Fig. 6g, h). In this study, we detected the expression of related genes in *MeCP2* knockdown xenografts. These results were consistent with the upregulation trend induced by the overexpression of *miR-22* (Fig. 6i). As in our previous study, *MeCP2* knockdown inhibited GC growth in vivo (Supplementary Fig. S6a–c). These in vitro and in vivo results suggest that *miR-22* could reduce the methylation of tumor suppressor genes and promote their expression by inhibiting SAM synthesis.

### Effect of SAM on GC cell growth and its correlation with *miR-22*, *MTHFD2*, and *MTHFR* expression

**Fig. 7:**
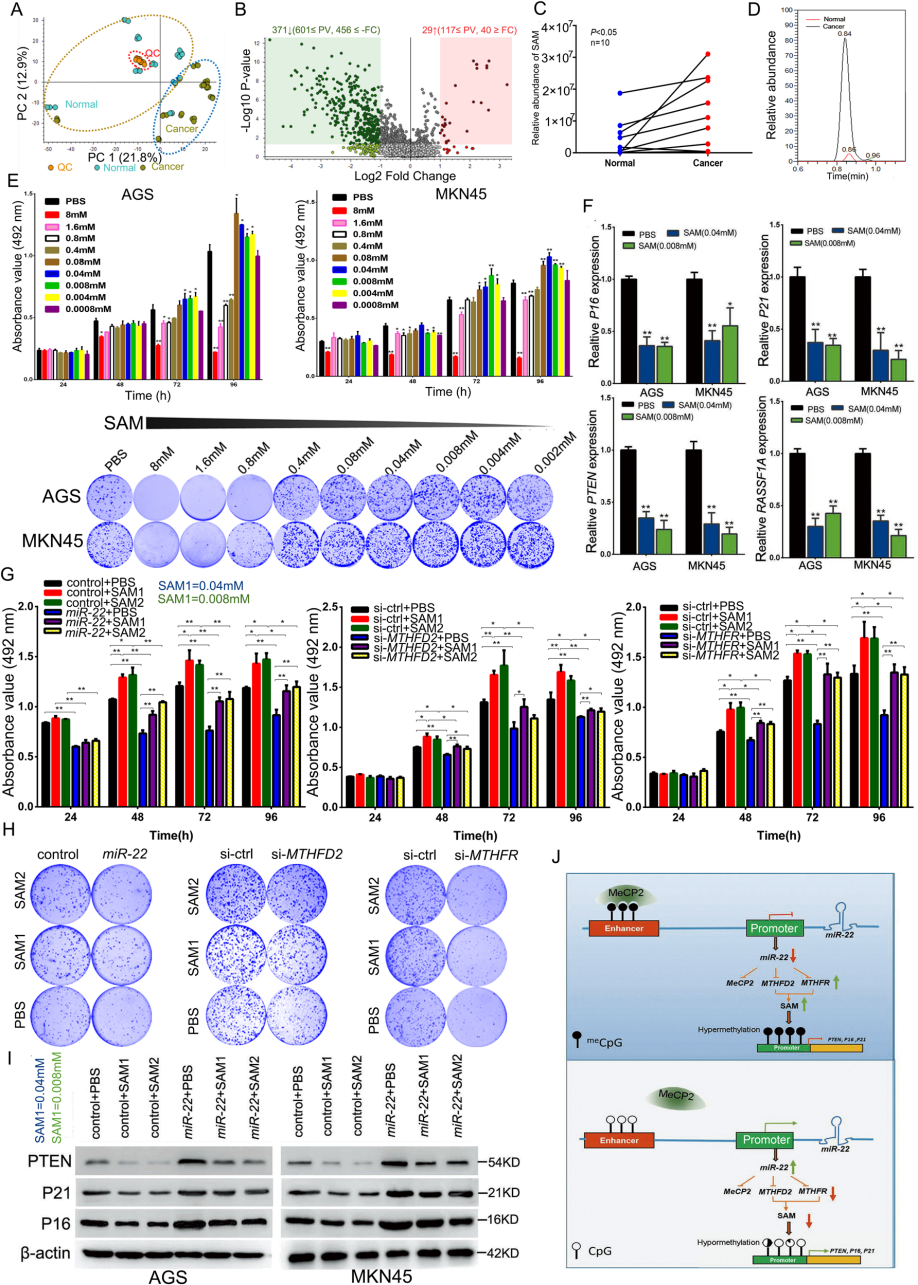
SAM promotes gastric cancer cell proliferation at low concentrations, and *miR-22* overexpression and MTHFD2, MTHFR knockdown rescues low SAM concentration-induced proliferation in GC cells. **a** PCA score plots derived from 10 paired clinical sample metabolomics assay data (positive ion mode, QC is short for quality control). **b** Volcano plot showing the metabolite variation trend in GC compared with normal samples. **c** SAM quantitative results for all samples. **d** SAM peak area for the paired clinical samples. **e** MTT (top) and colony formation (bottom) assays were performed to examine AGS and MKN45 cell proliferation after treatment with different concentrations of SAM. **f** *P16*, *PTEN*, *P21*, and *RASSF1A* mRNA levels in AGS and MKN45 cells treated with two concentrations of SAM (0.04 mM and 0.008 mM). **g** MTT assays was performed to measure the growth of MKN45 cells after treatment with low concentrations of SAM and *miR-22*, *MTHFD2* siRNA or *MTHFR* siRNA transfection. **h** colony formation assays were performed to measure the growth of MKN45 cells after treatment with low concentrations of SAM and *miR-22*, *MTHFD2* siRNA or *MTHFR* siRNA transfection. **i** PTEN, P21, and P16 protein expression was analyzed after treatment with low concentrations of SAM and *miR-22*. **j** Proposed model for the effect of MeCP2 on *miR-22* transcription, and *miR-22* suppressed GC cell proliferation by inducing an endogenous SAM deficit. The results are shown as mean±SD. *n* = 3, *p < 0.05, **p < 0.01, Student’s *t* test.

To understand the metabolic profiles and determine the SAM levels in normal and cancer tissues, a metabolomics assay was performed in 10 pairs of clinical samples. As shown in Fig. 7a, the principal component analysis indicated that the metabolic profiles of normal tissues were different from those of GC tissues. The expression of 371 metabolites increased, whereas that of 29 metabolites decreased (Fig. 7b). In addition, the relative quantification results showed that SAM was significantly higher in GC samples than in normal samples (nine increased, one decreased) (Fig. 7c, d). To analyze the effect of SAM on GC cell proliferation, we treated two cell lines with different concentrations of SAM and performed MTT and colony formation assays. Compared with phosphate-buffered saline (PBS) treatment, high concentrations of SAM (8, 1.6, 0.8 mM) significantly inhibited the proliferation of GC cells, but low concentrations (0.08, 0.04, 0.008, and 0.004 mM) promoted the proliferation activity (Fig. 7e). To verify the effect of low SAM concentrations on tumor suppressor expression, AGS and MKN45 cells were treated with SAM (0.04 and 0.008 mM). The qRT-PCR showed downregulation of *P16*, *RASSF1A*, *PTEN*, and *P21* with both SAM concentrations in AGS and MKN45 cells (Fig. 7f). As previously described, *miR-22* inhibited the endogenous SAM and affected the proliferation of cancer cells. We selected two SAM concentrations (0.04 and 0.008 mM) that promoted cell proliferation and verified that they reversed the inhibitory effects of *miR-22* overexpression and *MTHFD2* and *MTHFR* knockdown on cell proliferation (Fig. 7g,h, Supplementary Fig. S6d and S6e). In addition, SAM, at the same concentrations, also reversed the effects of *miR-22* overexpression on PTEN, P21, and P16 expression (Fig. 7i). These results suggest that SAM is a member of the molecular pathway through which *miR-22* mediates *MTHFD2*/*MTHFR* silencing and inhibits GC cell proliferation. MeCP2 binding with a high-level methylation enhancer led to the inhibition of *miR-22* in cancer cells. *miR-22* targets *MeCP2*, its own epigenetic regulator, thus forming a positive feedback loop. Two other *miR-22* targets, MTHFD2 and MTHFR, are key enzymes in folate metabolism. *miR-22* induces a deficit in the methyl donor SAM through a long-axis regulatory mechanism and ultimately inhibits the methylation of tumor suppressor genes (Fig. 7j).

## Discussion

*MiR-22*, a suppressor gene that contributes to the development of malignant tumors, inhibits tumor proliferation, invasion, and metastasis by mediating tumor growth status and energy supply^35^. However, it remains unclear whether the one-carbon metabolism is regulated by *miR-22*. We confirmed that *miR-22* is downregulated in GC, whereas *MiR-22* overexpression inhibits the proliferation of GC cells and induces apoptosis in GC cells. *MTHFD2* and *MTHFR* are novel targets of *miR-22*. MeCP2 was another target of *miR-22*, which was also supported by Fang^36^. The enhancer could promote gene expression over several Kbs^35,36^. The MeCP2-binding site located at an upstream enhancer of *miR-22* includes three methylated CpG sites. Our study revealed that *miR-22* is subject to epigenetic regulation by MeCP2 and is involved in the regulation of carbon metabolism in GC.

One-carbon metabolism is a metabolic network that includes nucleotide metabolism, maintenance of cellular redox status, lipid biosynthesis, and methylation metabolism^37^. Among these components, the folate and methionine cycles generate the universal methyl donor SAM for histone and DNA methylation, of which MTHFD2 and MTHFR are key enzymes. *MTHFD2* is one of the 50 most commonly overexpressed genes in cancer^31,37,38^. We found that MTHFD2 expression is higher in gastric tumor tissues and cancer cell lines, and demonstrated that *MTHFD2* silencing inhibits growth, arrests the cell cycle at G1/G0, and induces the apoptosis of gastric cells. Several previous studies have shown that *MTHFR* polymorphisms are significantly associated with an increased risk of lung, hepatocellular, and GC^39–41^. Our data show that downregulation of MTHFR leads to the inhibition of cell growth and G0/G1 cell cycle arrest and induces apoptosis. Our results suggest that MTHFD2 and MTHFR are involved in the development of GC.

SAM, the most common methyl group donor, is involved in tumor progression by regulating multiple cell processes, such as proliferation, differentiation, cell cycle regulation, and apoptosis. SAM levels are related to different effects on the regulation of cell processes^29,42–44^. SAM has been implicated as an antitumor player by affecting a variety of pathways, including methylation. For example, SAM induces the hypermethylation of the c-myc promoter and inhibits the growth of human GC cells^44^. However, in another study, SAM caused c-myc promoter hypermethylation, but did not change P16 methylation^43^. It is known that tumor suppressors have a hypermethylated promoter in cancer, but hypomethylation normal tissue. These findings drive the hypothesis that SAM could cause tumor suppressor hypermethylation in normal cells and drive tumorigenesis. Current evidence indicates that methionine, a substrate for SAM synthetase, is a metabolic dependency of tumor-initiating cells^45^. Moreover, cancer stem cells (CSCs) rely on SAM biosynthesis. Methionine restriction inhibits CSCs, and SAM supplementation rescues the effects of methionine restriction, at least partly^46^. Based on several sets of data discussed, SAM is the principal biological methyl donor synthesized in cells, which maintains chromatin methylation. In this study, we demonstrated that *miR-22* reduces *P16*, *RASSF1A*, and *PTEN* methylation by decreasing endogenous SAM (Figs. 6b–e). The direct effects of SAM on tumor cells show differential effects of high and low SAM concentrations (Fig. 7e). These effects are probably owing to the endogenously produced SAM, emerging as an endogenous tumor cell proliferation-producing factor. The cellular uptake at low concentrations of SAM is closer to the basal cellular levels than at high concentrations. Low-concentration uptake is beneficial for cancer cells to maintain endogenous methylation and against endogenous biochemical reactions such as demethylation^47,48^. Studies have also shown that low doses (0.1.1 mM) of SAM promote colon cancer cell line growth and inhibits proliferation at high doses (3 mM)^49^. At the same time, dietary intake of high concentrations of folic acid, an important molecule in carbon metabolism, can increase levels of serum folic acid, which can lead to DNA methylation of *P16*, *MLH1*, and *MGMT*^50^. Our study demonstrates that SAM reduces the expression of the tumor suppressor genes *P16*, *P21*, *PTEN*, and *RASSF1A*. Interestingly, *P21* inhibition is mainly regulated by histone methylation^51,52^. However, this does not contradict our conclusion that SAM deficit induced P21 upregulation. As described in our introduction, MTHFR catalyzes the reduction of methionine and 5-MTHF to SAM. A recent study reported that the methionine cycle flux specifically influences the epigenetic state of cancer cells and drives tumor initiation. Histone methylation is upregulated in SAM-treated cells and downregulated in *MTHFR* knockdown cells^45^. Other research findings indicate that dietary methionine influences therapy in mouse cancer models through one-carbon metabolism^53^. To confirm the effect of *miR-22* on one-carbon metabolism, we examined the changes in THF and SAM concentrations and found that *miR-22* prevented the synthesis of both substances and induced the expression of *P16*, *P21*, *PTEN*, and *RASSF1A*. SAM reversed the inhibition of *miR-22*, *MTHFD2*, and *MTHFR* knockdowns on cell proliferation and antagonized the expression of *P16*, *P21*, *PTEN*, and *RASSF1A* induced by *miR-22*, suggesting that *miR-22* is involved in the inhibition of SAM synthesis by targeting *MTHFD2* and *MTHFR*.

In conclusion, *miR-22* expression is inhibited by MeCP2. *MiR-22* targets *MTHFD2* and *MTHFR* to inhibit SAM synthesis and induces *P16*, *P21*, *PTEN*, and *RASSF1A* upregulation, which inhibits the proliferation of GC cells. This study suggests that the MeCP2 -*miR-22*-MTHFD2-MTHFR axis may be a therapeutic target for GC patients.

## Materials and methods

### GC tissues and cell lines

Paired GC and adjacent non-tumor gastric tissues were obtained from 36 patients who underwent surgical resection at the First Affiliated Hospital of Xi’an Jiaotong University and had not undergone previous surgery, radiotherapy, or chemotherapy. The tissue samples were immediately snap-frozen in liquid nitrogen until RNA extraction. Both tumor and non-tumor tissues were histologically confirmed. Informed consent was obtained from each patient, and the study was approved by the Institute Research Ethics Committee at Cancer Center, Xi’an Jiaotong University. Human GC AGS and MKN45 GC cell lines were obtained from the Cell Bank (Shanghai GeneChem Co., Ltd., Shanghai, China). The AGS and MKN45 cell lines were maintained in 1640 medium (1640; PAA Laboratories GmbH) supplemented with 10% fetal bovine serum (PAA Laboratories GmbH) and cultured in a humidified 5% CO_2_ incubator at 37 °C. The cell lines were transfected with Lipofectamine 2000 (Invitrogen, Carlsbad, CA, USA) following the manufacturer’s protocol.

### Animals

Five-week-old male BALB/c nude mice (Central Laboratory of Animal, Xi’an Jiaotong University Health Science Center) were bred under specific pathogen-free conditions. All animal experiments were approved by the Institutional Animal Care and Use Committee of Xi’an Jiaotong University and were performed according to the institution’s guidelines for laboratory animals. A total of 1 × 10^6^ *mir-22* or control lentivirus transfected with MKN45 cells were resuspended in 100 μL PBS and directly injected into the groin of each mouse. The tumor volumes were calculated using the following formula: *V* = *L* × (*W* × ½)^2^ (*L*: longest dimension, *W*: shortest dimension). After 40 days, the GFP activity of xenografts in mice was detected using a small animal imaging system. After the mice were killed, the xenografts were removed, and the next step was performed.

### Plasmid, siRNA, primers, lentivirus, and antibody

A human *miR-22* precursor was subcloned into pcDNA6.2 GW/eGFP, which was purchased from Invitrogen. The synthetic primary transcript of *miR-22* was designed into *Eco*RI and *Hin*dIII enzyme restriction sites to facilitate the cloning of vectors. The complementary sites in the 3′-UTR of *MeCP2*, *MTHFD2*, and *MTHFR* for *miR-22* were cloned into a pmirGLO vector at the *Sac*I and *Xho*I sites (Promega, Madison, USA). The PEGFP-C1 vector containing cDNA of *MeCP2* was purchased from GENEWIZ (Suzhou, China). The GFP-tagged *MeCP2* vector was only used to identify the MeCP2 valid domain that interacts with *miR-22*. The *MeCP2*, *MTHFD2*, and *MTHFR* overexpression vector and control (OE control) were purchased from GeneChem (Shanghai, China). The inhibitor of *miR-22-3p*, a small interfering RNA (siRNA) targeting *MeCP2*, *MTHFD2*, and *MTHFR*, was purchased from GenePharma. The *MiR-22* lentivirus overexpression vector was purchased from GeneChem (Shanghai, China).

### Clone formation assay

Equal numbers of cells were trypsinized, resuspended, and seeded at a density of 1000 cells/mL into six-well plates and incubated at 37 °C for 14 days. The colonies were stained with 0.1% crystal violet for 15 min and visualized by phase imaging.

### Flow cytometry assay

The cell cycle experiments were performed as follows: the cells were trypsinized, washed, and fixed with 70% ice-cold ethanol at 4 °C overnight. The cells were washed and suspended in 100 μl of 0.1 mg/mL RNase A and 100 μL of 0.05 mg/mL propidium iodide (PI) for 15 min at room temperature. The distribution of the cell cycle phase was examined by flow cytometry (FACSCalibur, BD Biosciences, CA, USA).

Apoptosis analysis was performed following the manufacturer’s instructions (Invitrogen, Carlsbad, CA, USA). The percentage of cell phase was examined by flow cytometry (FACSCalibur, BD Biosciences, CA, USA).

### Overlap PCR

The MBD deletion gene (*MeCP2*^ΔMBD^) was amplified twice by performing an overlap PCR with the following primers: AF (5′-CAAGCTTCGATGGCTGCCGCTGCTGCCGCTGCTCC-3′), AR (5′-TCTGTTCCCTCCTGCTAGGGCTATCCCTGATGATGGACCTCCTT-3′), BF (5′-AAGGAGGTCCATCATCAGGGATAGCCCTAGCAGGAGGGAACAGA-3′), and BR (5′-CGGGATCCTCAGCTCACTCTCTCGGTGACAGGG-3′). The primers AF and BR contained *Hin*dIII and *Bam*HI restriction sites, respectively, which were used to clone them into a PEGFP-C1 vector. The TRD deletion gene (*MeCP2*^ΔTRD^) was constructed using the same protocol, but different overhang primers: AR (5′-TCCTTGACTTCGATGCTGACGGTGCCCTCGCTGGTAGCGGCTTTG-3′) and BF (5′-CAAAGCCGCTACCAGCGAGGGCACCGTCAGCATCGAAGTCAAGGA-3′). The TRD partial deletion gene (*MeCP2*^ΔTRD+NLS^) was constructed based on *the MeCP2*^ΔTRD^ vector using the following primers: AR (5′-TAGGAATGGCCTGGGGGTCGGCCTCAGCCTTCCTGCCCTCGCTGGTAGCGGCTT-3′) and BF (5′-CCCCCAGGCCATTCCTAAAAAGAGGGGCAGGAAGACCGTCAGCATCGAAGTCAA-3′).

### Chromatin immunoprecipitation

ChIP was performed as previously described^3^. In brief, AGS and MKN45 cells were harvested and crosslinked with 1% formaldehyde for 15 min at room temperature. The lysate was immunoprecipitated with Dynal magnetic beads (Invitrogen) and antibodies against MeCP2, IgG, and GFP. The DNA was isolated, and 10% of the IP lysate was used as input. ChIP products were assayed by qPCR and PCR gel analysis. Two standard methods were utilized to normalize the ChIP-qPCR data: the percent input method and the fold enrichment method. The input and IP products were amplified by PCR, followed by electrophoresis analysis.

### Immunofluorescence

Cells were seeded on eight-well chamber slides. After 48 h, the cells were fixed with 4% paraformaldehyde for 15 min. After washing with PBS, the cells were permeabilized for 20 min with PBST (0.5% Triton X-100 in PBS) at room temperature and then washed with PBS. The cells were blocked with PBSB (0.1% Triton X-100 and 4% BSA in PBS) for 2 h at room temperature and then incubated with the primary antibody overnight at 4 °C. After washing three times with PBS, the cells were incubated with the secondary antibody for 2 h at room temperature in the dark. After washing three times with PBS, the slides were incubated with DAPI (1 µg/mL in 1× PBS) for 10 min at room temperature in the dark. After washing with PBS three times, the slides were observed under a fluorescence microscope.

### Bisulfite sequencing PCR assay

The levels of CpG methylation were measured with a BSP assay using a Bisulfite Conversion Kit (Active Motif, 55016) per the manufacturer’s instructions. In brief, genomic DNA was extracted using a genomic DNA extraction kit (Omega, D3396). The DNA was subjected to C/T conversion using a bisulfite reagent. We amplified the converted DNA with methylation-specific primers, designed by the MethPrimer and used TA cloning after a sequencing assay to measure the methylation level.

### Luciferase report assay

The *miR-22* enhancers were amplified from the whole genome of a Hek-293 cell; enhancer1 and enhancer2 were constructed into a pGL3-Promoter Vector (Promega, E1761). The serial architecture enhancer was constructed by overlap PCR. The AGS and MKN45 cells were transfected with pGL3-Control Vector (Promega, E1741), pGL3-Promoter Vector, pGL3-Promoter Vector-E1, pGL3-Promoter Vector-E2, pGL3-Promoter Vector-E1+E2, and pGL3-Basic Vector (Promega, E1751). After 24 h, the luciferase activity was measured using a microplate system.

A dual-luciferase assay was used to verify the relationship between *miR-22* and *MeCP2*, *MTHFD2*, and *MTHFR*. The Hek-293 cells were co-transfected with *miR-22* expression vector and pmirGLO–*MeCP2*, -*MTHFD2*, and -*MTHFR* 3′UTR-WT or pmirGLO–*MeCP2*, -*MTHFD2*, and -*MTHFR* 3′UTR-MUT reporter vectors. After 48 h of transfection, the luciferase activity of each group was tested using the Dual-Glo Luciferase Assay kit (Promega) following the manufacturer’s protocol.

### Public clinical data sets analysis

Data regarding *MTHFD2* and *miR-22* expression were downloaded from the UCSC Xena (https://xenabrowser.net)^54^. Type of data: gene expression RNAseq, Version: 2017-10-13, platform: IlluminaHiSeq_RNASeqV2. Type of data: miRNA mature strand expression RNAseq, version: 2017-09-08, platform: IlluminaHiSeq_miRNASeq. The Hi-C data, including A549 (GSE92819), Caki2 (GSE105465), H1-ESC (GSE52457), NCIH460 (GSE105725), T47D (GSE105697), and K562 (GSE63525) were downloaded from the GEO data set^55,56^. TAD borders were analyzed using the TADtool^57^.

## Supplementary information

Patient characteristics and clinico pathologic

Condition and method of mobile phase in HPLC-MS assay

All primer sequence, antibody and siRNA information

Dataset 1

Dataset 2A

Dataset 2B

Dataset 3

supplementary figure 1

supplementary figure 2

supplementary figure 3

supplementary figure 4

supplementary figure 5

supplementary figure 6

Supplementary figure legends
