# Supplementary material for: MiR-22, regulated by MeCP2, suppresses gastric cancer cell proliferation by inducing a deficiency in endogenous S-adenosylmethionine
Source: Oncogenesis. 2020 Nov 10;9(11):99. doi: 10.1038/s41389-020-00281-z (PMC7652948; doi:10.1038/s41389-020-00281-z)
Supplement: Supplementary file 1 — Patient characteristics and clinico pathologic [file 41389_2020_281_MOESM1_ESM.docx]

| Characteristics | Number of cases |
| --- | --- |
| Age |  |
| ≥60 years | 23 |
| <60 years | 16 |
| Gender |  |
| Male | 28 |
| Female | 11 |
| Histology |  |
| Well | 13 |
| Moderate | 15 |
| poor | 11 |
| Tumor size |  |
| <50 mm | 19 |
| ≥50 mm | 20 |
| Lymph node metastasis |  |
| Yes | 24 |
| No | 15 |

Patient characteristics and clinicopathologic
