# Supplementary material for: MiR-22, regulated by MeCP2, suppresses gastric cancer cell proliferation by inducing a deficiency in endogenous S-adenosylmethionine
Source: Oncogenesis. 2020 Nov 10;9(11):99. doi: 10.1038/s41389-020-00281-z (PMC7652948; doi:10.1038/s41389-020-00281-z)
Supplement: Supplementary file 2 — Condition and method of mobile phase in HPLC-MS assay [file 41389_2020_281_MOESM2_ESM.docx]

**Condition of mobile phase in HPLC-MS assay**

| Time  (min) | Flow Rate  (mL/min) | Compositio  nA(%) | Compositio  nB(%) |
| --- | --- | --- | --- |
| 0.00 | 0.100 | 95.0 | 5.0 |
| 10.00 | 0.100 | 95.0 | 5.0 |
| 13.00 | 0.100 | 0 | 100.0 |
| 16.00 | 0.100 | 0 | 100.0 |
| 17.00 | 0.100 | 95 | 5.0 |
| 18.00 | 0.100 | 95 | 5.0 |

**Method**

High-performance liquid phase mass spectrometry analysis

Metabolite extraction was processed according as previously described^[1]^. The deferent groups’ adherent cells were digested with trypsin to obtain a single cell. The suspensions were adjusted to the same concentration according to the cell count. Part of the cell suspensions (equal volume) were retained for the Western blot experiments using actin as internal control to strictly ensure that the same experiment system is used. The cells’ metabolic activity was terminated with a quenching solution (60% methanol, 0.85% AMBIC), which was cooled to −40°C. The cells were collected by centrifugation and resuspended with 100% methanol (-80°C), followed by a snap-freeze in a liquid nitrogen. The metabolites were extracted from the quenched cells using two 100% methanol extractions followed by a single water extraction. The metabolite extract was condensed in a nitrogen blowing concentrator. A HPLC–MS/MS analysis was performed using the Vion IMS Qtof system (Waters, USA). ACUITY UPLC-HSS T3 Colum (186003539, Waters, dimension: 1 mm × 100 mm, particle: 1.8 μm) was used as the stationary phase; different proportions of solvent A (0.1% formic acid) and solvent B (methyl alcohol) were used as the mobile phase solvents. The samples were ionized in a positive ion mode. Mass spectrometry was performed using the following operating parameters: capillary voltage: 1.00 kV, source temperature: 100°C, desolvation temperature: 500°C, cone gas flow rate: 50 L/h, desolvation gas flow rate: 800 L/h, scan setting: low mass: 50 m/z, high mass: 2 000 m/z, and scan time: 0.200 s.

**Gradient elution condition of Q Exactive Plus metabolomics assay**

| Time  [min] | % A | % B |
| --- | --- | --- |
| 0 | 98 | 2 |
| 1 | 98 | 2 |
| 11 | 2 | 98 |
| 15 | 2 | 98 |
| 15.1 | 98 | 2 |
| 18 | 98 | 2 |

**Method**

Q-Exactive Plus metabolomics of tissue samples

The metabolomics assay was performed in Thermo Fisher Scientific (Shanghai, China). The aqueous phase extraction of metabolites in tissue samples was performed as follows: a 50 mg sample was placed into prechilled methanol/water (1:1), the sample was homogenized using a Qiagen Tissuelyser (25 Hz speed, 5 min/cycle), the sample was centrifuged (16 000 g, 10 min), the supernatant was dried in a Savant vacuum concentrator (time: 180 min, temp: 45℃, mode: V-AQ), and the dry residue was re-dissolved in 12 0μL of methanol/water (1:1) (UHPLC conditions: HPLC system: Thermo Dionex Ultimate 3000, column: Hypersil Gold aQ (2.1×100 mm, 1.9 µm), mobile phase: A: 0.1FA H2O (Pos) or H2O (Neg), B: CH3OH; flow rate: 0.3 mL/min, column temp: 30°C; injection volume: 2 μL; MS conditions: MS system: Q-Exactive Plus, ionization mode: ± ESI, spray voltage: +3800 or −3200, vaporize temperature: 400°C, capillary temperature: 300°C, sheath gas: 40 arb, Aux gas: 10 arb, resolution: 140,000 (FWHM)). The Compound Discoverer data analysis platform was used to process and analyze the data.

1 Sellick CA, Hansen R, Stephens GM, Goodacre R, Dickson AJ. Metabolite extraction from suspension-cultured mammalian cells for global metabolite profiling. *Nature protocols* 2011; 6: 1241-1249.
