# Supplementary material for: MiR-22, regulated by MeCP2, suppresses gastric cancer cell proliferation by inducing a deficiency in endogenous S-adenosylmethionine
Source: Oncogenesis. 2020 Nov 10;9(11):99. doi: 10.1038/s41389-020-00281-z (PMC7652948; doi:10.1038/s41389-020-00281-z)
Supplement: Supplementary file 3 — All primer sequence, antibody and siRNA information [file 41389_2020_281_MOESM3_ESM.pdf]

All primer sequence	
qRT-PCR primer	oligonucleotides
mir-22-3p-RT	GTCGTATCCAGTGCGTGTCGTGGAGTCGGCAATTGCACTGGATACGACACAGTTC
mir-22-3p-F	ATCCAGTGCGTGTCGTG
mir-22-3p-R	TGCTAAGCTGCCAGTTGAA
MeCP2-F	GCCGAGAGCTATGGACAGCA
MeCP2-R	CCAACCTCAGACAGGTTTCCAG
MTHFD2-F	GGCAGTTCGAAATGAAGCTGTT
MTHFD2-R	GCCAACCAGGATCACACTCA
MTHFR-F	AGCTGGGCCTGAAGAACATC
MTHFR-R	ACTGCGTAGTTGAAGCCTCC
P16-F	CAACGCACCGAATAGTTACG
P16-R	CAGCTCCTCAGCCAGGTC
PTEN-F	GGGTCTGAGTCGCCTGTCA
PTEN-R	CCGTGTTGGAGGCAGTAGAAG
P21-F	CACTCCAAACGCCGGCTGATCTTC
P21-R	TGTAGAGCGGGCCTTTGAGGCCCTC
RASSF1A-F	GTTCACCTGCCACTACCGC
RASSF1A-R	ACCGTCCTTGTTCAAGCTCA
BSP primer	oligonucleotides
miR-22-enhancer-F	GGGCCAGTGGTTTCACTGTAG
miR-22-enhancer-R	CCCCCAAGACTTCCTGTTGAA
P16-F	TTTTGTTTTTTAAATTTTTTGGAGG
P16-R	AAACCCAATCCTCCTTCCTTAC
PTEN-F	TTGTTATTATTTTTAGGGTTGGGAA
PTEN-R	CTAAACCTACTTCTCCTCAACAACC
RASSF1A-F	GTTTGTGATAGAAATTAAGGGGGTT
RASSF1A-R	AACCAAATAAAAACCAAAAAAATACC
Enhancer clone primer	oligonucleotides
E1F	CGGATCCCTAGGCCTTGGCGTCACGGTG
E1R	GCGTCGACGGCGCCGGGGGGATTCCATG
E2F	CGGATCCCTAGGCCTTGGCCGGTCACGG
E2R	GCGTCGACTGAGACGGAGTCTCACTCTG
OL-R	GTGACGCCAAGGCCTAGGGCGCCGGGGGGATTCC
OL-F	GGAATCCCCCCGGCGCCCTAGGCCTTGGCGTCAC
ChIP primer	oligonucleotides
ChIP-enhancer-F	CACAGTGACATCTGGAGGC
ChIP-enhancer-R	GAGGCCTGGTGTTTTGGG
H3K27Ac/p300-chip-F	ATTCCTCAAGGGGTCTTAGTTC
H3K27Ac/p300-chip-R	TTTTCCTAAGTGTCTTTATGGG
GAPDH-ChIP-F	TACTAGCGGTTTTACGGGCG
GAPDH-ChIP-F	TCGAACAGGAGGAGCAGAGAGCGA

Antibody information		
Antibody	Cat No	company
MeCP2	ab2828	Abcam
MTHFD2	ab151447	Abcam
MTHFR	GTX100535	GeneTex
GFP	ab290	Abcam
P16	80772	Cell Signaling Technology
P21	2947	Cell Signaling Technology
PTEN	9188	Cell Signaling Technology
CDK4	12790	Cell Signaling Technology
H3K27Ac	ab4729	Abcam
RNA polymeraseII	ab817	Abcam
p300	ab14984	Abcam

siRNA sequence		
gene	oligonucleotides	
	sense（5'-3'）	antisense（5'-3'）
MeCP2 siRNA1	GCUUCCCGAUUAACUGAAATT	UUUCAGUUAAUCGGGAAGCTT
MeCP2 siRNA2	GCUUAAGCAAAGGAAAUCUTT	AGAUUUCCUUUGCUUAAGCTT
MTHFD2 siRNA1	GGAAGGAGCAGCAGUCAUUTT	AUGACUGCUGCUCCUUCCTT
MTHFD2 siRNA2	GAGCAGUUGAAGAAACAUATT	UAUGUUUCUUCAACUGCUCTT
MTHFR siRNA1	GGGUGAAAACAUCACCAAUTT	AUUGGUGAUGUUUUCACCCTT
MTHFR siRNA2	GCAAUUGCCCUGUAACUUATT	UAAGUUACAGGGCAAUUGCTT
Control siRNA	UUCUCCGAACGUGUCACGUTT	ACGUGACACGUUCGGAGAATT
