# Supplementary figures and images for: MiR-22, regulated by MeCP2, suppresses gastric cancer cell proliferation by inducing a deficiency in endogenous S-adenosylmethionine
Source: Oncogenesis. 2020 Nov 10;9(11):99. doi: 10.1038/s41389-020-00281-z (PMC7652948; doi:10.1038/s41389-020-00281-z)

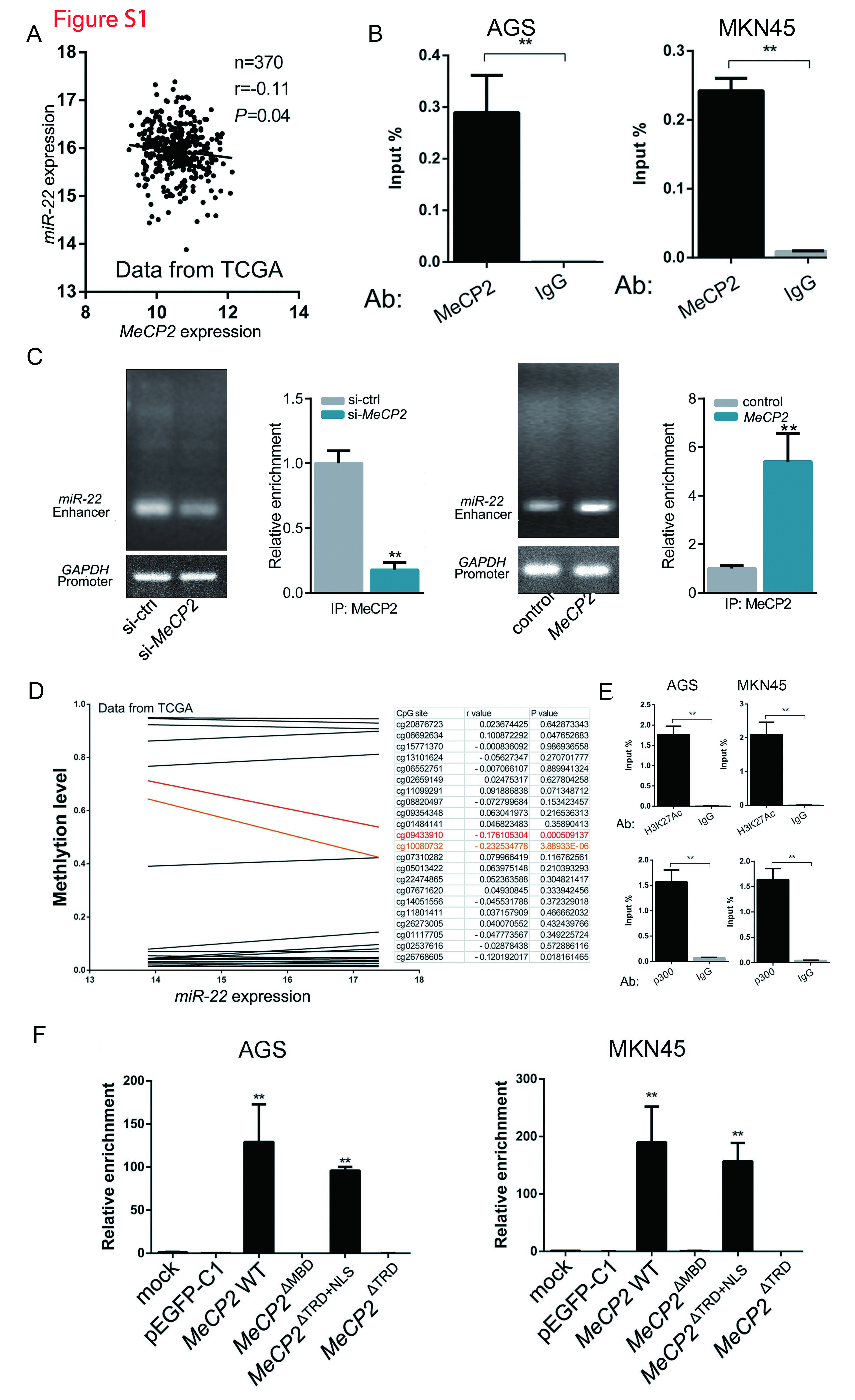

Supplement: Supplementary file 8 — supplementary figure 1 [file 41389_2020_281_MOESM8_ESM.jpg]

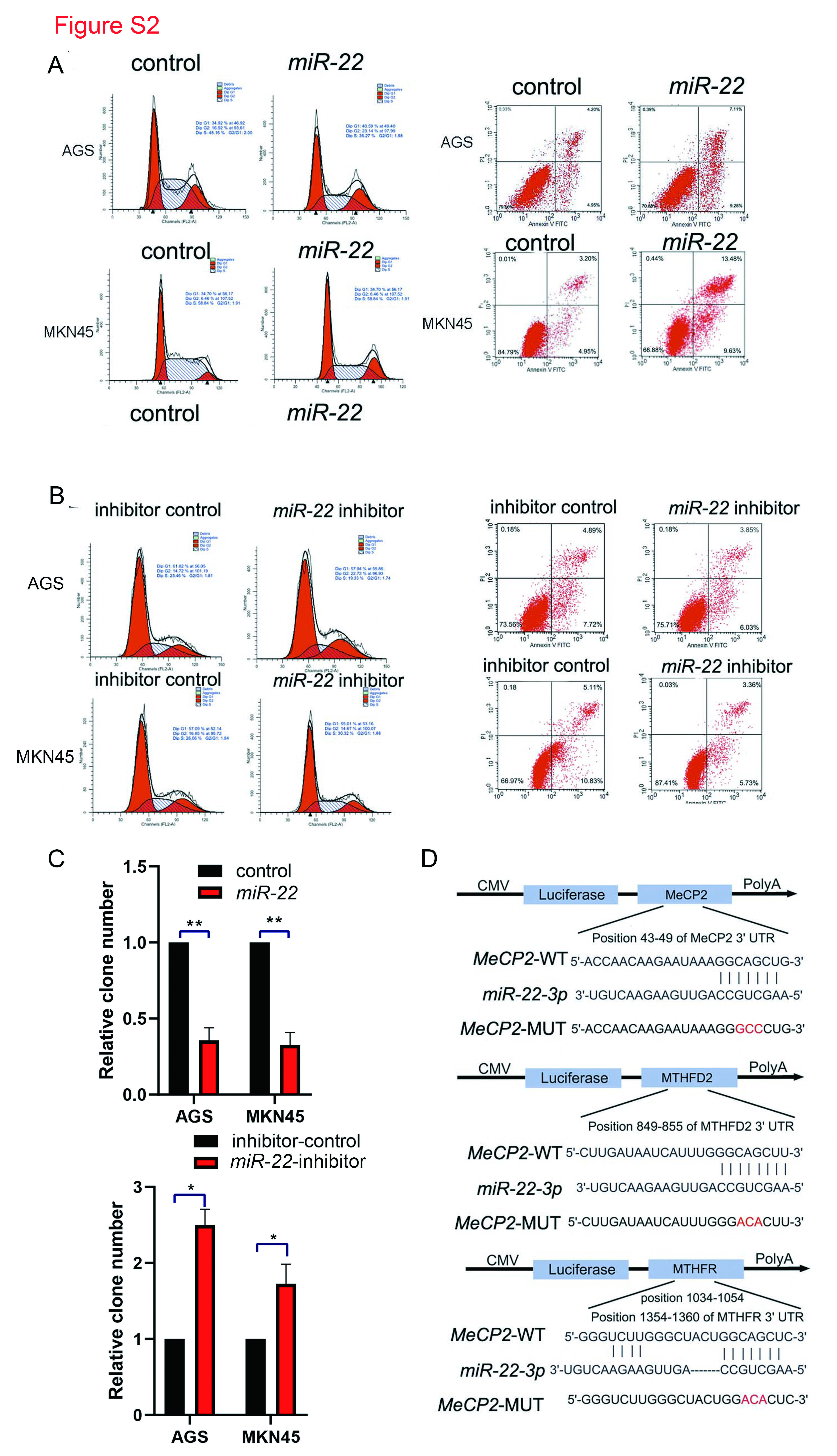

Supplement: Supplementary file 9 — supplementary figure 2 [file 41389_2020_281_MOESM9_ESM.jpg]

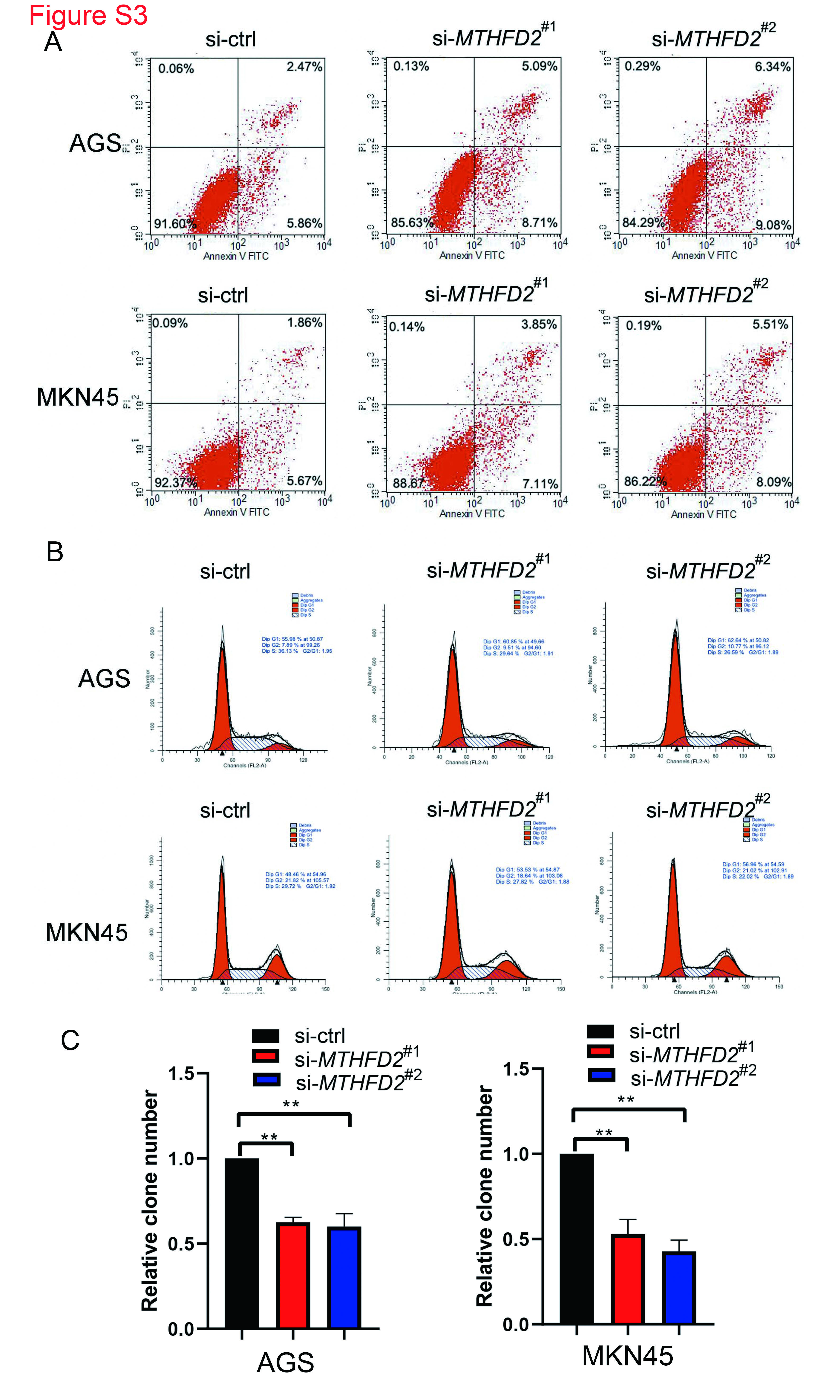

Supplement: Supplementary file 10 — supplementary figure 3 [file 41389_2020_281_MOESM10_ESM.jpg]

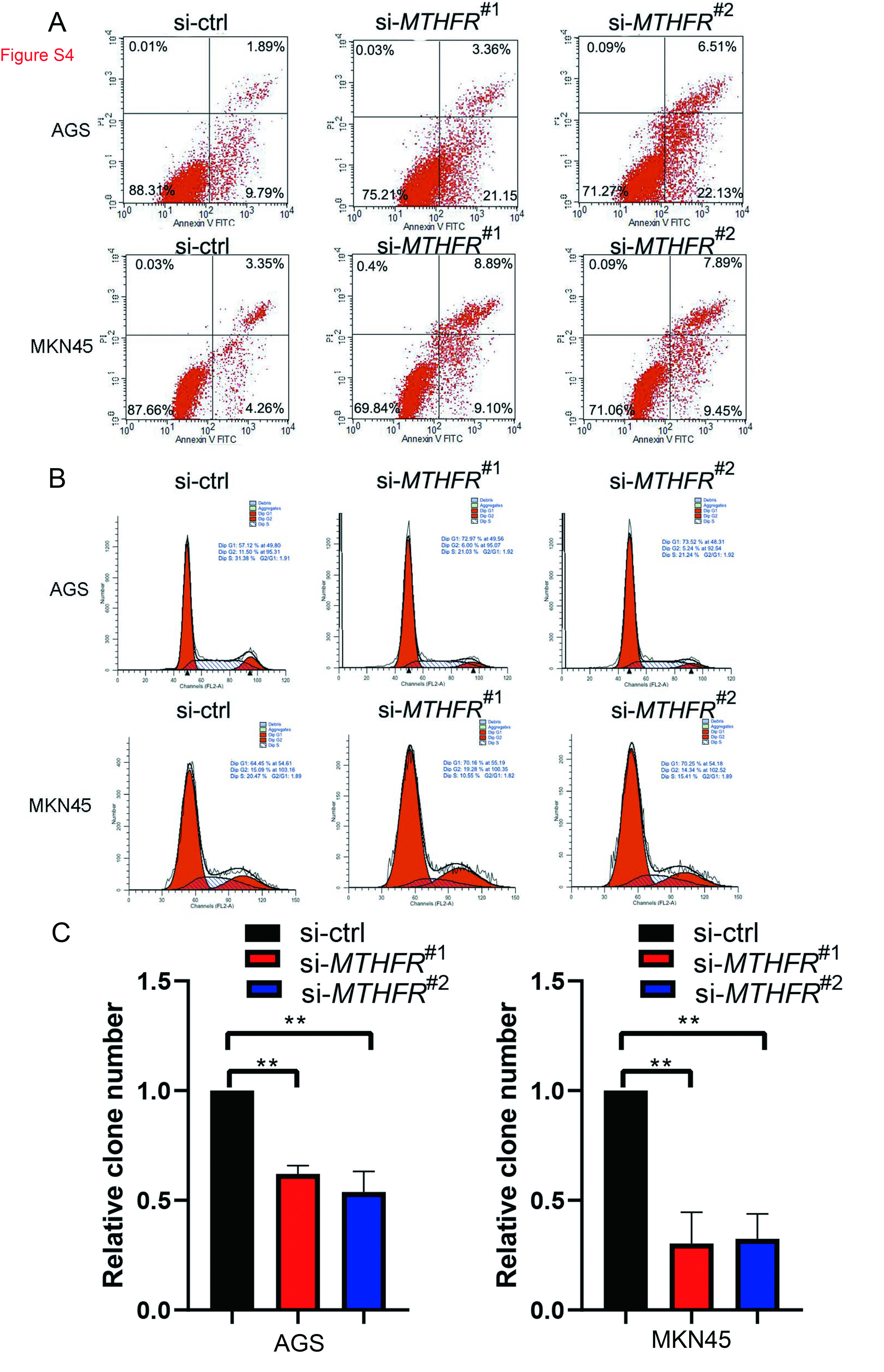

Supplement: Supplementary file 11 — supplementary figure 4 [file 41389_2020_281_MOESM11_ESM.jpg]

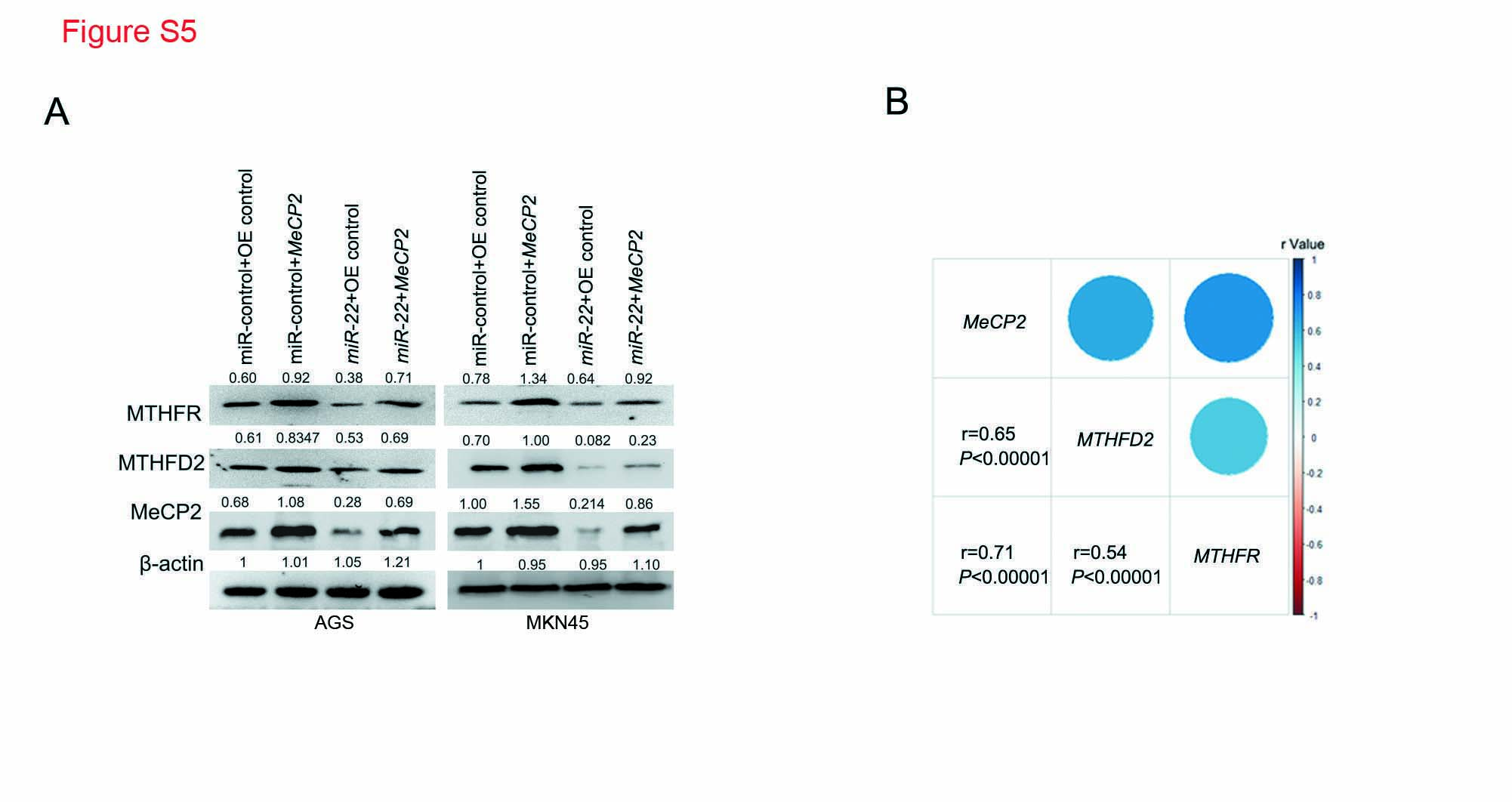

Supplement: Supplementary file 12 — supplementary figure 5 [file 41389_2020_281_MOESM12_ESM.jpg]

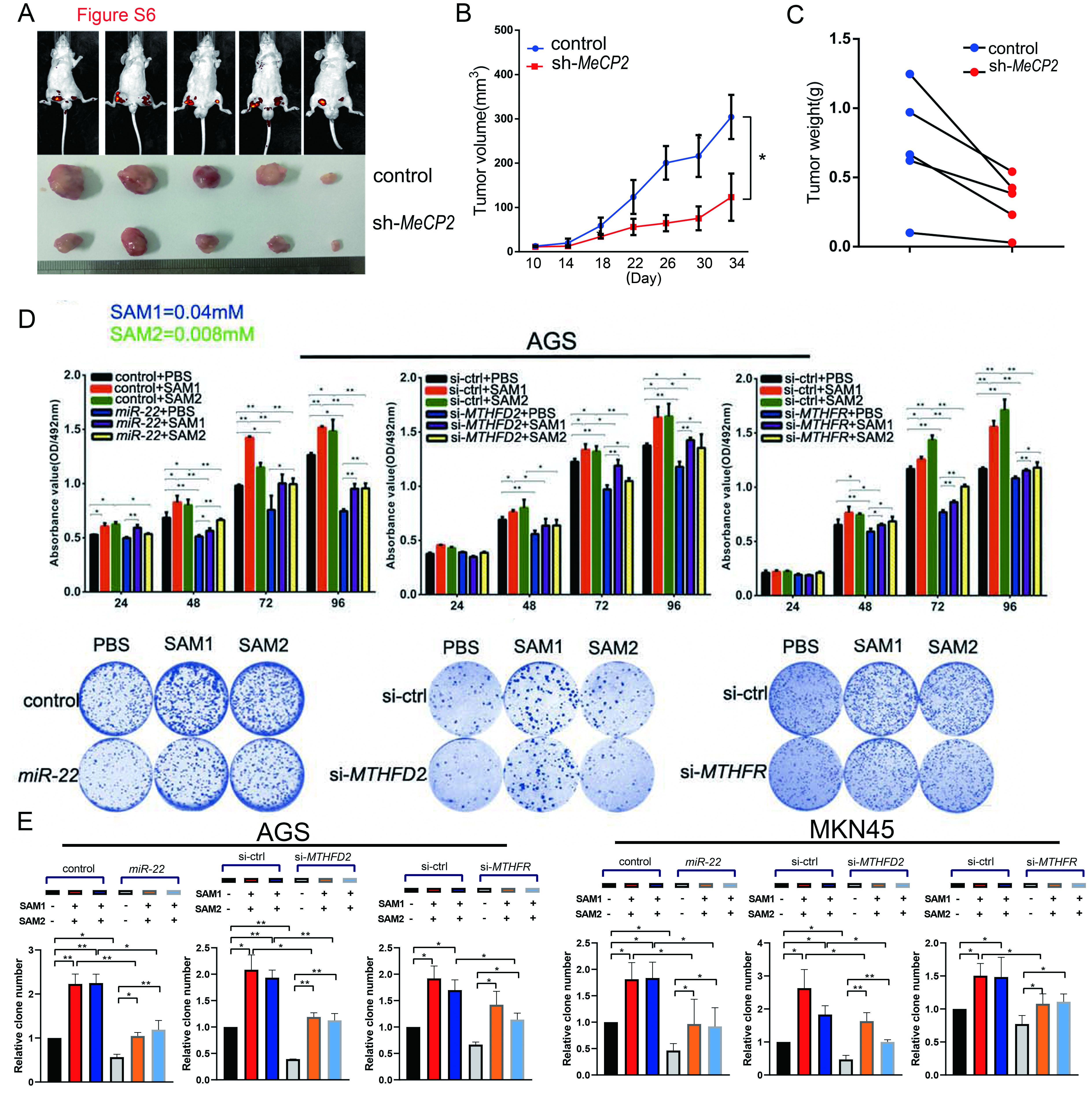

Supplement: Supplementary file 13 — supplementary figure 6 [file 41389_2020_281_MOESM13_ESM.jpg]
