# Supplementary material for: MiR-22, regulated by MeCP2, suppresses gastric cancer cell proliferation by inducing a deficiency in endogenous S-adenosylmethionine
Source: Oncogenesis. 2020 Nov 10;9(11):99. doi: 10.1038/s41389-020-00281-z (PMC7652948; doi:10.1038/s41389-020-00281-z)
Supplement: Supplementary file 14 — Supplementary figure legends [file 41389_2020_281_MOESM14_ESM.docx]

**Supplementary figure Legends**

**Supplementary Fig. 1** (A): TCGA data analysis showed a significant correlation between *MeCP2* and *miR-22*. The correlation was tested by Pearson analysis on the condition that *p* < 0.05. (B): ChIP-qPCR was used to capture the *miR-22* enhancer region with MeCP2 antibodies in GC cells. (C): ChIP-PCR and ChIP-qPCR assays were used to capture the miR-22 enhancer region with MeCP2 antibodies in *MeCP2* knockdown and overexpression AGS cells. Simultaneous ChIP analysis of poly II A binding to *the GAPDH* promoter as an internal reference. (D): The correlation between *miR-22* and ten CpG sites upstream and downstream of the two sites that MeCP2 binds. Pearson’s correlation analysis was used to test the correlations. (E): Analysis of the binding of H3K27ac and P300 to enhancers according to ChIP-qPCR. (F): The domain responsible for binding to *the miR-22* enhancer. Different mutations of *the MeCP2* vector were transfected into GC cells. The interaction was verified by ChIP-qPCR analysis. The results are shown as mean ± SD. n=3, **p* < 0.05, ***p* < 0.01.

**Supplementary Fig. 2:**

(A): Raw flow cytometry data for *miR-22*-overexpressing AGS and MKN45 cells. (B): Raw flow cytometry data for *miR-22* inhibitor-transfected AGS and MKN45 cells. (C): Relative quantification of colony formation assay 14 days after transfection. (D): The target sites of *miR-22* that bind to *MeCP2*, *MTHFD2*, and *MTHFR* 3’UTRs, and the WT and MUT sequences were inserted into the pmirGLO dual luciferase reporter vector. The results are shown as mean ± SD. n=3, **p* < 0.05, ***p* < 0.01.

**Supplementary Fig. 3:** Raw flow-cytometry data showing the effect of *MTHFD2* knockdown on MKN45 and AGS cell apoptosis (A), cell cycle (B), and relative quantification of colony formation assay(C). The results are shown as mean ± SD. n=3, **p* < 0.05, ***p* < 0.01.

**Supplementary Fig. 4:** Raw flow-cytometry data showing the effect of *MTHFR* knockdown on MKN45 and AGS cell apoptosis (A), cell cycle (B), and relative quantification of colony formation assay(C). The results are shown as mean ± SD. n=3, **p* < 0.05, ***p* < 0.01.

**Supplementary Fig. 5:** (A): AGS and MKN45 cells were co-transfected with *MeCP2* and *miR-22*, and western blotting was performed to assess MeCP2, MTHFD2, and MTHFR protein levels. (B): The TCGA data were used to analyze the correlation between the expression of *MeCP2*, *MTHFD2*, and *MTHFR*. The correlation was tested by Pearson analysis.

**Supplementary Fig. 6**: (A): *In vivo* small animal image and morphology of excised tumors from nude mice. (B) Growth curves of tumors. (C): Tumors weight on day 34 after the initial injection. (D): MTT and colony formation assays were performed to measure the growth of AGS cells after treatment with low concentrations of SAM, *miR-22*, *MTHFD2* siRNA*, or MTHFR* siRNA transfection. (E): Relative quantification of colony formation assay of AGS and MKN45 cells after treatment with low concentrations of SAM and *miR-22*, *MTHFD2* siRNA*, or MTHFR* siRNA transfection. The results are shown as mean ± SD. n=3, **p* < 0.05, ***p* < 0.01.
